# Prediction of esophageal cancer risk based on genetic variants and environmental risk factors in Chinese population

**DOI:** 10.1186/s12885-024-12370-y

**Published:** 2024-05-16

**Authors:** Haiyan Liu, Keming Li, Junfen Xia, Jicun Zhu, Yifan Cheng, Xiaoyue Zhang, Hua Ye, Peng Wang

**Affiliations:** 1 Department of Epidemiology and Statistics, College of Public Health, Zhengzhou University, Zhengzhou City, 450001 Henan Province China; 2 Henan Key Laboratory of Tumor Epidemiology and State Key Laboratory of Esophageal Cancer Prevention & Treatment, Zhengzhou University, Zhengzhou City, 450052 Henan Province China; 3 Zhengzhou Center for Disease Control and Prevention, Zhengzhou City, 450042 Henan Province China; 4 Office of Health Care, the Third Affiliated Hospital of Zhengzhou University, Zhengzhou City, 450052 Henan Province China; 5 Department of Pharmacy, the First Affiliated Hospital of Zhengzhou University, Zhengzhou City, 450052 Henan Province China

**Keywords:** Esophageal cancer, Meta-analysis, Single-nucleotide polymorphism, Weighted genetic risk score, Risk prediction model

## Abstract

**Background:**

Results regarding whether it is essential to incorporate genetic variants into risk prediction models for esophageal cancer (EC) are inconsistent due to the different genetic backgrounds of the populations studied. We aimed to identify single-nucleotide polymorphisms (SNPs) associated with EC among the Chinese population and to evaluate the performance of genetic and non-genetic factors in a risk model for developing EC.

**Methods:**

A meta-analysis was performed to systematically identify potential SNPs, which were further verified by a case-control study. Three risk models were developed: a genetic model with weighted genetic risk score (wGRS) based on promising SNPs, a non-genetic model with environmental risk factors, and a combined model including both genetic and non-genetic factors. The discrimination ability of the models was compared using the area under the receiver operating characteristic curve (AUC) and the net reclassification index (NRI). The Akaike information criterion (AIC) and Bayesian information criterion (BIC) were used to assess the goodness-of-fit of the models.

**Results:**

Five promising SNPs were ultimately utilized to calculate the wGRS. Individuals in the highest quartile of the wGRS had a 4.93-fold (95% confidence interval [CI]: 2.59 to 9.38) increased risk of EC compared with those in the lowest quartile. The genetic or non-genetic model identified EC patients with AUCs ranging from 0.618 to 0.650. The combined model had an AUC of 0.707 (95% CI: 0.669 to 0.743) and was the best-fitting model (AIC = 750.55, BIC = 759.34). The NRI improved when the wGRS was added to the risk model with non-genetic factors only (NRI = 0.082, *P* = 0.037).

**Conclusions:**

Among the three risk models for EC, the combined model showed optimal predictive performance and can help to identify individuals at risk of EC for tailored preventive measures.

**Supplementary Information:**

The online version contains supplementary material available at 10.1186/s12885-024-12370-y.

## Background

Esophageal cancer (EC) remains a public health issue globally. EC was the seventh most common cancer in incidence and ranked as the sixth leading cause of cancer-related mortality worldwide in 2020 [1]. In China, new cases of EC and related deaths account for 53.70% and 55.35% of the world’s totals, respectively [1, 2]. Moreover, the overall 5-year survival rate for patients with EC in China remains dismal at only 15–25% [3]. Like other cancers, early diagnosis can contribute to a dramatically improved 5-year survival rate for patients with EC [4]. Epidemiological studies have shown that relevant variables, such as smoking and alcohol consumption, are risk factors for EC, and striking sex and age disparities also exist [5, 6]. In addition, the existence of various genetic variants is closely associated with susceptibility to EC [7, 8].

To improve early detection of EC, a promising approach is to establish a risk prediction model that incorporates well-recognized risk factors to identify high-risk individuals in advance. Furthermore, ethnic differences in either genetic factors or histologic subtypes deserve full consideration. EC includes esophageal squamous cell carcinoma (ESCC) and esophageal adenocarcinoma (EAC). In China, ESCC is predominant.

As an effective tool to improve risk stratification, risk prediction models have been developed based on a combination of genetic and non-genetic factors for various malignancies, such as breast cancer [9] and colorectal cancer [10]. In 2008, Yokoyama et al. [11] constructed a prediction model for EC by incorporating a single-nucleotide polymorphism (SNP) and four individual risk factors. The results showed that compared with conventional screening protocols, the positive predictive value of endoscopy for the top 10% of risk in the model was increased by approximately 1.7%. However, one SNP cannot adequately represent the genetic variants related to EC, and the study was conducted only in the Japanese male population. In addition, Chang et al. [12] developed a prediction model for ESCC in Chinese population by including 25 SNPs and 4 non-genetic factors. However, inclusion of a large number of SNPs hampers cost-effectiveness. In 2018, Dong et al. [13] developed a risk model for EAC among people of European ancestry by including 23 genetic variants and several epidemiologic factors. The conclusions of these studies regarding whether it is essential to incorporate genetic factors into risk models for EC were inconsistent due to the different genetic backgrounds of the populations included. To the best of our knowledge, studies including genetic variants in risk prediction models for EC are still limited for the Chinese population to date. Genetic predisposition, as a well-established risk indicator of EC, warrants further research to clarify its value in predicting the risk of developing EC [14].

In this study, a meta-analysis was performed to comprehensively identify potential SNPs that may predispose individuals to EC in Chinese population. A case-control study was carried out to verify the associations of these SNPs with EC, followed by construction of risk prediction models based on a panel of well-established risk factors and promising SNPs to provide an effective tool for identifying individuals at high risk.

## Methods

### Meta-analysis for selecting candidate SNPs

The meta-analysis was conducted according to the Preferred Reporting Items for Systematic Reviews and Meta-Analyses (PRISMA) statement.

#### Search strategy

To identify SNPs related to EC, a comprehensive literature search was performed using the following online databases up to July 1, 2020: PubMed, EMBASE, Web of Science, Cochrane Library, CNKI (Chinese), WanFang (Chinese), and CBM (Chinese). The following search terms were used: (risk factors) AND (esophageal OR esophagus) AND (neoplasm OR cancer OR tumor OR neoplastic OR carcinoma OR adenocarcinomas OR malignancy OR malignancies OR neoplasia) AND (single nucleotide polymorphism OR SNP OR variant OR variation OR polymorphism) AND (Chinese OR China).

#### Inclusion and exclusion criteria

The eligibility criteria were as follows: (1) studies on associations between SNPs and EC risk; (2) studies for which odds ratios (ORs) and 95% confidence intervals (CIs) were available; (3) studies for which the genotype distribution in the controls was in accordance with Hardy-Weinberg equilibrium (HWE); and (4) case-control or cohort-designed study. The exclusion criteria were as follows: (1) not original studies (reviews, meta-analyses, letters, and abstracts); (2) fewer than three studies for one SNP; (3) studies for which the sample size of cases or controls was less than 10; and (4) studies for which the minor allele frequency was less than 1% in the control group. For studies based on the same population, we selected only the study with the most informative data.

#### Data extraction and quality assessment

The following data were extracted independently by two authors: the first author, year of publication, study region, cancer type, gene, SNP, distribution of genotypes in case and control groups, type of controls, genotyping method, and quality control. Any discrepancies were resolved through discussion with a third investigator. The Newcastle-Ottawa Scale (NOS) was used to evaluate the quality of the studies. We rated the quality as 0–9, with scores of 5–6 and 7–9 being judged to represent moderate and high quality, respectively.

### A case-control study for verifying candidate SNPs

#### Subjects

In total, 500 EC patients and 500 controls were enrolled for the current study. All cases were obtained from a third-level grade A hospital in Henan Province, China, in 2018 and confirmed by pathology reports. Controls were randomly selected from participants in a cardiovascular disease epidemiological survey simultaneously conducted in Henan Province and were frequency-matched to cases by sex. The exclusion criteria for patients and controls were as follows: (1) patients with EC who had a history of another tumor; (2) controls who experienced health problems, including tumors and esophagus-related diseases.

Basic information of the subjects with EC was retrieved from clinical records, and the controls were administered a professionally designed questionnaire that assessed information regarding non-genetic factors. Individuals who had smoked at least one cigarette every 1–3 days for more than six months were considered smokers. Individuals who had drunk alcohol at least once a week for more than six months were considered drinkers. This study was approved by the Institutional Review Board of Zhengzhou University, and all participants provided informed consent.

#### Genotyping and quality control

A GeneJET Whole Blood Genomic DNA Purification Mini Kit was used to extract DNA. Improved multiplex ligation detection reaction (iMLDR™) was used to genotype SNPs in the case group. ABI3730XL sequencer (AppliedBiosystems, U.S.A) and GeneMapper 4.0 were used for sequencing and identification of genotypes, respectively. Genotyping in the control group were performed via DNA sequencing. All DNA samples were successfully genotyped.

For quality control, agarose gel electrophoresis was applied for each sample before genotyping. The quality of genotyping was assessed by using negative quality control and repeated genotyping of 3% of the samples randomly selected. Moreover,10% of the samples in the case group were further genotyped by using DNA sequencing to verify the concordance of the two methods.

### Construction of risk prediction models for esophageal cancer

Data were randomly split into a training set (60%, 301 cases and 299 controls) for developing risk prediction models and a verification set (40%, 199 cases and 201 controls) for evaluating the resulting models.

Three models containing different variables were developed: a genetic model with genetic markers only; a non-genetic model fit with environmental risk factors, including smoking, alcohol consumption, and family history of esophageal cancer; and a combined model including both genetic and non-genetic predictors.

Promising SNPs verified in the case-control study were utilized to calculate the weighted genetic risk score (wGRS). The genetic model was then constructed using this wGRS [15]. Logistic regression was employed to develop non-genetic and combined models.

The wGRS is estimated as follows:

The genetic score of single SNP was calculated based on the OR of the risk allele and the frequency of genotype in Chinese population (Chinese Han in Beijing, CHB).

Genetic score (W) = (1-p)^2^+2p(1-p)OR + p^2^OR^2^ (p is the risk allele frequency).

AA = 1/W; AB = OR/W; BB = OR^2^/W (A is the non-risk allele; B is the risk allele; AA, AB, and BB refer to the SNP genotype).

wGRS = SNP_1_×SNP_2_×SNP_3_×SNP_4_……SNP_n_ (Missing value set to 1).

### Statistical analysis

In the meta-analysis, ORs with 95% CIs were used for assessment of associations between genetic variants and EC risk. Statistical heterogeneity was evaluated by means of the Cochran *Q*-test and *I*^*2*^ statistic. A fixed-effects model (Mantel-Haenszel) was applied if the *P* value was ≥ 0.10 or *I*^*2*^ was ≤ 50%; otherwise, a random-effects model (DerSimonian-Laird) was applied. Begg’s test and Egger’s test were conducted to examine publication bias.

Unconditional logistic regression was performed to evaluate associations between genetic variants and EC risk in this case-control study. The chi-square test of goodness of fit was employed to analyze whether the distribution of genotypes in the control group matched HWE. For significant SNPs, the false-positive report probability (FPRP) was calculated to verify the authenticity of the summary results [16, 17]. The default value of the FPRP critical value was 0.5, and the prior probabilities were set to 0.25, 0.1, and 0.01. The attributable risk percentage (*ARP*) and population attributable risk percentage (*PARP*) were calculated to evaluate the epidemiological effect of each SNP.

Receiver operating characteristic (ROC) curves and the net reclassification index (NRI) were utilized to evaluate the discrimination of the different models with the area under the ROC curve (AUC), sensitivity, specificity, positive likelihood ratio, negative likelihood ratio, and accuracy rate. Comparison of AUCs was further performed by using DeLong’ test [18]. The Akaike information criterion (AIC) and Bayesian information criterion (BIC) were adopted to determine the goodness-of-fit of the models.

R software (version 4.2.2), MedCalc (version 20.027), SPSS (version 26.0), and Stata statistical software (version 15.1) were used in this study. Statistical significance was determined at α = 0.05, and all *P* values for statistical significance were two-sided.

## Results

### Main findings from the meta-analysis

The screening procedure is summarized in online Additional file 1: Figure S1. After duplicate exclusion (*n* = 2 865), title or abstract screening (*n* = 3 336), and full-text review (*n* = 336), a total of 100 articles (149 SNP-related studies) were ultimately included in the subsequent analysis (online Additional file 3: Supplementary References). If two populations or SNPs were present in one article, we considered it to be two independent studies. The studies included 48 654 cases and 58 373 controls, involving 29 SNPs located in 22 genes. The number of datasets for each SNP ranged from 3 to 11, with the most widely studied SNP being *ALDH2* rs671. More details of the SNPs are provided in online Additional file 2: Table S1 and Additional file 1: Figure S2.

Twelve SNPs significantly decreased or increased the risk of EC (*P53* rs1042522, *CYP1A1* rs1048943, *ADH1B* rs1229984, *ERCC2* rs13181, *NQO1* rs1800566, *MMP13* rs2252070, *PLCE1* rs2274223, *CDKN1A* rs2395655, *CYP2E1* rs3813867, *TERT* rs401681, *CYP1A1* rs4646903, and *IL23R* rs6682925) (online Additional file 2: Table S2). Specifically, six SNPs (*CYP1A1* rs1048943, *ADH1B* rs1229984, *ERRCC2* rs13181, *MMP13* rs2252070, *PLCE1* rs2274223, and *CYP2E1* rs3813867) were significant under all 5 genetic models. The most significant association with EC risk was observed for *CYP1A1* rs1048943 under the homozygous model (OR = 2.44, 95% CI: 1.79 to 3.33). For the 12 significant SNPs indicated above, FPRP was the best for 12/12, 12/12 and 9/12 at the 0.25, 0.1 and 0.01 levels, respectively (online Additional file 2: Table S3), which suggests that the findings are relatively reliable. The top three SNPs for ARP were *CYP2E* rs3813867 (65.87%), *CYP1A1* rs1048943 (59.02%), and *ADH1B* rs1229984 (55.16%). Moreover, the top three SNPs for PARP in the controls and CHB were the same as those for ARP. PARP for each SNP between the control group and CHB was similar, suggesting the controls to be representative (online Additional file 2: Table S4). Additionally, the findings from publication bias assessments provided little indication of publication bias except for *ERCC2* rs13181 and *NQO1* rs1800566.

### Characteristics of the population

The detailed characteristics of the study subjects are shown in Table 1. There was no significant difference in sex between the patients and control subjects because of the frequency-matched design. The mean age was significantly older in the case group (63.00 ± 8.33) than in the control group (46.80 ± 11.55). As expected, compared with the controls, the EC patients were more likely to smoke, drink alcohol, and have a family history of esophageal cancer.

**Table 1:** Baseline characteristics of participants in the case-control study

Variables	Cases	Controls	t/χ^2^	*P* value
	(*n* = 500)	(*n* = 500)		
Age, *y*, (Mean ± SD)	63.00 ± 8.33	46.80 ± 11.55	25.418	**< 0.001**
Sex, *n* (%)				
Male	363(72.6)	363(72.6)	< 0.001	1.000
Female	137(27.4)	137(27.4)		
Smoking status, *n* (%)				
Yes	222(44.4)	173(34.6)	10.047	**0.002**
No	278(55.6)	327(65.4)		
Drinking status, *n* (%)				
Yes	194(38.8)	148(29.6)	9.403	**0.002**
No	306(61.2)	352(70.4)		
Family history of esophageal cancer, *n* (%)				
Yes	78(15.6)	10(2.0)	57.616	**<0.001**
No	422(84.4)	490(98.0)		

NOTE: *χ*^*2*^ test was performed for categorical variables and Student *t* test was for continuous variables. SD: standard deviation

### Evaluation and verification of SNPs in the case-control study

A total of 14 SNPs were evaluated, including 12 SNPs identified in the previous meta-analysis and another two SNPs, namely, *MTHFR* rs1801133 and *ALDH2* rs671, included in a large number of studies in the above meta-analysis and considered to be significant in reviews [19, 20]. Finally, five promising SNPs (*P53* rs1042522, *MTHFR* rs1801133, *PLCE1* rs2274223, *ALDH2* rs671, and *ADH1B* rs1229984) were validated as EC susceptibility loci (online Additional file 2: Table S5 and Table S6). For *P53* rs1042522 and *ADH1B* rs1229984, the best-fitting genetic model was recessive and the ORs were 0.69 (95% CI: 0.48 to 1.00) and 1.78 (95% CI: 1.08 to 2.94), respectively. For *MTHFR* rs1801133, *PLCE1* rs2274223, and *ALDH2* rs671, the best-fitting genetic model was the dominant model, with ORs of 0.41 (95% CI: 0.26 to 0.65), 1.93 (95% CI: 1.37 to 2.71), and 2.42 (95% CI: 1.65 to 3.56), respectively. For these 5 promising SNPs, FPRP was the best for 5/5, 5/5 and 3/5 at the 0.25, 0.10 and 0.01 levels, respectively (online Additional file 2: Table S7).

### Weighted genetic risk score (wGRS)

**Fig. 1:**
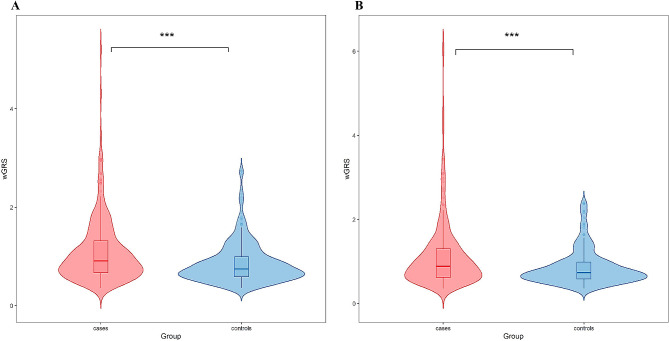
The distribution of wGRS in the case and control groups. (**A**) in the training set; (**B**) in the validation set. wGRS, weighted genetic risk score. ***P < 0.001

Details regarding the calculation of wGRS are described in online Additional file 2: Table S8. The wGRS was significantly greater in the patients than in the controls (Fig. 1). Next, we assessed the association between wGRS and EC risk (based on the quartile distribution in the controls), and found increased ORs across quartiles of wGRS (*P* for trend < 0.001 in the training set; *P* for trend = 0.005 in the validation set). The results showed that in the training set, individuals in only the highest quartile of wGRS had a 4.93-fold (95% CI: 2.59 to 9.38) increased risk of EC compared with those in the lowest quartile. In the validation set, a significantly increased risk was also observed only for the highest quartile (OR = 3.12, 95% CI: 1.53 to 6.36) (Table 2).

**Table 2:** Association of wGRS with the risk of esophageal cancer

wGRS	Training set			Validation set		
	OR (95%CI)	*P* value	*P* for trend	OR (95%CI)	*P* value	*P* for trend
Lowest	1.00(Reference)	-		1.00(Reference)	-	
Second	1.59(0.79,3.19)	0.194		1.01(0.46,2.23)	0.974	
Third	1.50(0.77,2.91)	0.235		1.45(0.68,3.11)	0.335	
Highest	**4.93(2.59,9.38)**	**< 0.001**	**< 0.001**	**3.12(1.53,6.36)**	**0.002**	**0.005**

NOTE: Based on the quartile distribution in the controls. Adjusted for age, smoking, alcohol consumption, and family history of esophageal cancer. wGRS, weighted genetic risk score. Training set: 301 cases and 299 controls; Validation set: 199 cases and 201 controls

**Table 3:** Evaluation of predictive performance and goodness of fit of risk prediction models

Indicators	Training set			Validation set		
	Genetic	Non-genetic	Combined	Genetic	Non-genetic	Combined
AUC	0.618	0.650	0.707	0.609	0.612	0.669
AUC 95%CI	(0.578,0.657)	(0.610,0.688)	(0.669,0.743)	(0.559,0.657)	(0.563,0.660)	(0.620,0.715)
Youden index	0.201	0.241	0.323	0.215	0.213	0.289
Sensitivity (%)	48.50	42.19	69.44	56.78	42.21	56.28
Specificity (%)	71.57	81.94	62.88	64.68	79.10	72.64
Accuracy (%)	60.00	62.00	66.17	60.75	60.75	64.50
Positive likelihood ratio	1.71	2.34	1.87	1.61	2.02	2.06
Negative likelihood ratio	0.72	0.71	0.49	0.67	0.73	0.60
AIC	805.41	776.78	750.55	538.84	530.50	511.80
BIC	814.21	785.58	759.34	546.82	538.49	519.78

NOTE: The genetic model was based on wGRS; the non-genetic model included non-genetic factors which were seen in the text; the combined model included both wGRS and non-genetic factors. AUC: area under the curve; AIC: Akaike information criterion; BIC: Bayesian information criterion; wGRS: weighted genetic risk score

### Construction and evaluation of risk prediction models

In the training set, the genetic model was constructed based on wGRS. The equation of the non-genetic model was as follows: Y_1_ = 1/(1 + EXP(-(-0.236-0.584 × _1_ + 2.038 × _2_ + 1.392 × _3_))) (X_1_, smoking; X_2_, family history of esophageal cancer; X_3_, the interaction of smoking and alcohol consumption). The combined model was expressed as follows: Y_2_ = 1/(1 + EXP(-(-1.110 + 0.908 × _1_-0.558 × _2_ + 1.976 × _3_ + 1.393 × _4_))) (X_1_, wGRS; X_2_, smoking; X_3_, family history of esophageal cancer; X_4_, the interaction of smoking and alcohol consumption) (online Additional file 2: Table S9).

**Fig. 2:**
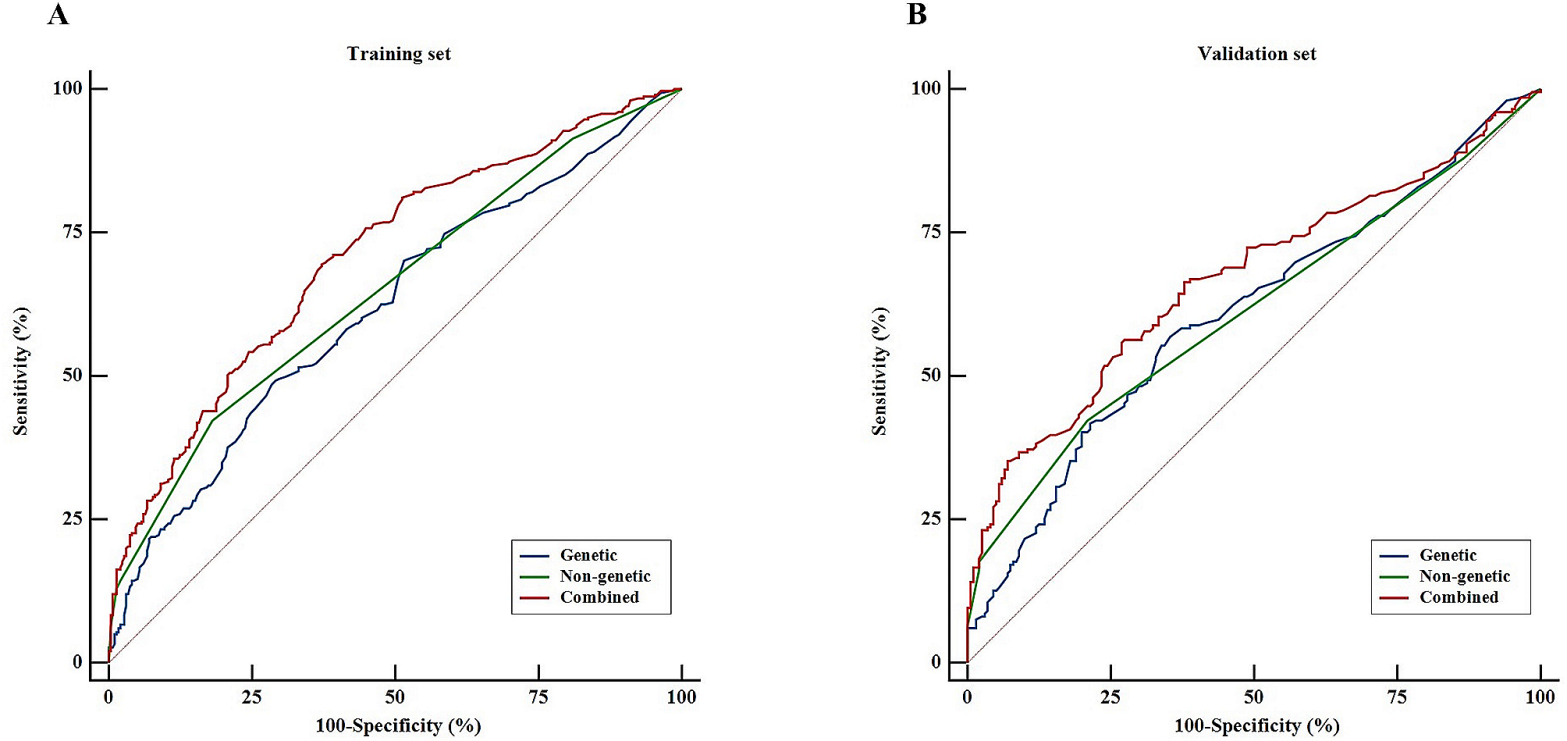
Receiver operating characteristic curves for risk prediction models of esophageal cancer. (**A**) the three models were constructed in the training set; (**B**) these models were verified in the validation set

We evaluated the discriminative ability of the models. The non-genetic model achieved moderate accuracy in distinguishing EC patients from controls, with an AUC of 0.650 (95% CI: 0.610 to 0.688). The model containing the wGRS alone had a relatively lower AUC of 0.618 (95% CI: 0.578 to 0.657). When comparing the two AUCs, no statistical significance was found (Delong’s test, *P* = 0.301). However, with the addition of wGRS, the AUC for the non-genetic model significantly increased from 0.650 to 0.707 (Delong’s test, *P* < 0.001). Overall, the combined model was superior to the other models with genetic or non-genetic parameters alone (Fig. 2; Tables 3 and 4). As shown in Table 3, the combined model had a sensitivity of 69.44%, a specificity of 62.88%, and an accuracy of 66.17%.

Based on the NRI, the prediction effect of the combined model was significantly greater than that of the model with non-genetic parameters alone in both the training and validation sets (training set: NRI = 0.082, *P* = 0.037; validation set: NRI = 0.076, *P* = 0.033). When comparing the combined and genetic models, the NRI significantly improved only in the training set (training set: NRI = 0.122, *P* = 0.001; validation set: NRI = 0.075, *P* = 0.225) (Table 4). According to the AIC and BIC, the combined model was selected as the best fitting model (AIC = 750.55, BIC = 759.34) (Table 3). Overall, the model incorporating both genetic and non-genetic factors showed optimal predictive performance.

The predictive performance of these models was then evaluated by using another independent validation set. A similar discrimination ability was observed, which indicated that the models had rosy stability.

**Table 4:** Comparison of different esophageal cancer risk prediction models

Model comparison	Difference of AUC	Z^a^	*P* value^a^	NRI	Z^b^	*P* value^b^
Training set						
Genetic vs. non-genetic	0.032(-0.028,0.091)	1.034	0.301	0.041	0.755	0.450
Genetic vs. combined	0.089(0.048,0.130)	4.256	< 0.001	0.122	3.234	0.001
Non-genetic vs. combined	0.058(0.030,0.086)	4.067	< 0.001	0.082	2.082	0.037
Validation set						
Genetic vs. non-genetic	0.004(-0.072,0.079)	0.091	0.927	-0.002	0.020	0.984
Genetic vs. combined	0.060(0.007,0.113)	2.223	0.026	0.075	1.214	0.225
Non-genetic vs. combined	0.057(0.021,0.092)	3.084	0.002	0.076	2.128	0.033

NOTE: The difference of AUC was analyzed using Delong’s test. NRI, net reclassification improvement.

^a^ represents the z-statistic and *P* value from Delong’s test;

^b^ represents the z-statistic and *P* value from NRI analysis

## Discussion

In this study, a meta-analysis approach was used to identify potential SNPs related to EC risk in Chinese population, and a case-control study was designed to verify the associations of these SNPs with EC risk. A total of three models were effectively constructed and evaluated. The results suggested that the combined model was preferable to the other models, which further supports that the addition of multiple genetic variants may provide reliable value in EC risk prediction.

In the meta-analysis, although the results of some SNPs were consistent with those of previous meta-analyses or a genome-wide association study (GWAS) [21, 22], there were some inconsistencies [23, 24]. For instance, a previous meta-analysis [24] revealed that *CYP1A1* rs4646903, which was significant in our meta-analysis, may not affect susceptibility to EC in Asian populations, while another meta-analysis [22] revealed that this statistically increasing risk was observed in the population from North China. These discrepant findings may be partly explained by differences in genetic susceptibility and environmental risk factors among diverse populations. Thus, to lessen the influence of different genetic backgrounds, our meta-analysis was conducted only in Chinese population.

Among the five promising SNPs used in the models, *ADH1B* rs1229984 and *ALDH2* rs671 are involved in ethanol metabolism [25, 26]. The rs1229984 C allele and rs671 A allele can result in accumulation of acetaldehyde [27, 28]. Among individuals with a combination of the two risk alleles, the level of *N*^*2*^-ethylidene-dG in the DNA of leukocytes from alcoholics was significantly increased, which enhanced DNA damage, leading to an elevated risk of EC [29]. The rs2274223 polymorphism in *PLCE1* affects esophageal carcinogenesis by enhancing the inflammatory response and upregulating phospholipase C epsilon mRNA, protein, and enzyme activity [30]. In addition, for *P53* rs1042522 and *MTHFR* rs1801133, associations with EC risk may vary among different populations. The vital polymorphism *P53* rs1042522, encoding proline or arginine, is located at codon 72 of exon 4 [31]. Several studies have reported an approximately twofold increase in the risk of EC in individuals with the rs1042522 CC genotype [32, 33], while other studies have shown that the GG genotype was a risk marker for human papillomavirus-associated EC [34, 35]. In our case-control study, the rs1042522 CC genotype reduced the risk of EC. Peng et al. also provided evidence that the CC genotype might be a risk factor for EC susceptibility in southern China but not in northern China [36]. Moreover, some studies have shown that the *MTHFR* rs1801133 TT genotype can increase the risk of EC [37, 38], while the rs1801133 T allele was showed to decrease EC risk in another study conducted in Henan Province, China [39]. There are several possible explanations for these different findings. The gene product of *MTHFR* is a central enzyme involved in folate metabolism, and the level of folate intake may influence the risk of EC associated with this polymorphism [40, 41]. In another study [42], the rs1801133 polymorphism increased EC risk, but the association disappeared after stratification by folate consumption. Additionally, the frequency of rs1801133 also differs by ethnicity [40].

Previous risk prediction models for EC were mostly based on non-genetic factors [14, 43–47], and easy-to-obtain variables were included in a standardized manner without any extra costs. However, for such a complex etiological disease, the actual predictive efficacy of environmental factors alone has not been completely established. In terms of numerous genetic variants, many studies [48, 49] on other cancers have reported that the predictive ability improved after adding genetic information to a model developed with non-genetic factors. For EC risk, Chang et al. [12] calculated the wGRS through the use of 25 SNPs and added the wGRS to the model with 4 non-genetic factors (sex, age, smoking status, and drinking status), with an elevated AUC ranging from 0.639 to 0.709. In another study, Dong et al. [13] used 23 GWAS-based SNPs to generate polygenic risk score (PRS) and found that individuals in the highest quartile had a more than 2-fold greater risk of developing EAC than those in the lowest quartile. However, Dong et al. noted that adding the PRS to a risk prediction model with non-genetic factors did not greatly improve its clinical use. Given that genetic predisposition is widely recognized as a well-established risk factor for EC, we constructed and evaluated risk prediction models with various combinations of genetic or non-genetic factors. Our findings provide supporting evidence that the addition of genetic predisposition significantly enhances performance in predicting EC risk.

There are several strengths of this study. First, meta-analysis was applied to comprehensively screen SNPs only in the Chinese population, avoiding the influence of different ethnicities. Second, the number of SNPs included in our risk models was relatively less than that in previous studies incorporating genetic variants [12, 13], which can improve cost-effectiveness. Third, when assessing whether there was improvement in discrimination after adding a new promising maker, the NRI was used to evaluate the degree of prediction increment in addition to AUC. The NRI especially focuses on the change in the number of individuals correctly discriminated by the new model compared to the old model, which can help to optimize limited resources. The goodness-of-fit of the model was evaluated using the AIC and BIC. After examining both the goodness-of-fit and predictive ability, the combined model was ultimately considered the optimal model in our study. Furthermore, the environmental risk predictors included in our models, such as smoking and alcohol consumption, were modifiable, which could enhance the awareness of adherence to healthy lifestyles.

Nevertheless, several limitations merit consideration. First, as mentioned previously, different genotyping methods were used in the case and control groups, which may bias the results. However, to minimize this bias, 10% of the samples from the cases were further genotyped by DNA sequencing, which was used for the controls, with consistent results. Second, external validation of our models was not conducted given the limited data availability, which included genetic data available outside of the present study. Third, genetic variants display regional and population differences, and our study constructed the wGRS associated with the risk of EC in the Chinese population through a case-control study, which may weaken the generalization of this wGRS to other racial or ethnic groups. Moreover, we must note that other related effect modifiers, such as the consumption of hot food and preserved vegetables, were not taken into consideration in our models; as such, they were not available for the study population. To address these issues, more comprehensive investigations should be performed when data are available.

## Conclusions

In summary, three risk prediction models were developed based on various combinations of the wGRS or environmental risk factors. The results indicated that the combined model including both genetic and non-genetic factors showed the optimal predictive performance for EC risk, which can help to identify individuals with an increased risk of EC for individualized prevention from early stages in life. Further studies on external validation and cost effectiveness are needed to verify the practical feasibility of the model.

### Electronic supplementary material

Below is the link to the electronic supplementary material.

**Additional file 1: Figure S1.** Flow chart of literature selection in the meta-analysis. **Figure S2**. Distribution of studies included in mete-analysis by province in China. (docx)

**Additional file 2:** **Table S1.** SNPs identified from the meta-analysis. **Table S2.** Associations of genetic variants with esophageal cancer risk in the meta-analysis. **Table S3.** Heterogeneity test and evaluation of reliability for genetic variants significantly associated with esophageal cancer risk. **Table S4.** Epidemiological effect estimation for the relationship between genetic variants and esophageal cancer. **Table S5.** Associations of 14 candidate SNPs with risk of esophageal cancer in the case-control study. **Table S6.** Associations of genotypes of 14 candidate SNPs with esophageal cancer risk. **Table S7.** False positive report probability of 5 promising SNPs. **Table S8.** Risk score for each promising SNP. **Table S9.** Construction of non-genetic and combined models. (docx)

**Additional file 3: Supplementary References.** (docx).

## Data Availability

The datasets used and/or analysed during the current study are available from the corresponding author on reasonable request.

## Abbreviations

EC: Esophageal cancer
ESCC: Esophageal squamous cell carcinoma
EAC: Esophageal adenocarcinoma
SNP: Single-nucleotide polymorphism
HWE: Hardy-Weinberg equilibrium
OR: Odds ratio
CI: Confidence interval
wGRS: Weighted genetic risk score
FPRP: False-positive report probability
*ARP*: Attributable risk percentage
*PARP*: Population attributable risk percentage
ROC: Receiver operating characteristic
NRI: Net reclassification index
AUC: Area under ROC curve
AIC: Akaike information criterion
BIC: Bayesian information criterion
CHB: Chinese Han in Beijing
GWAS: Genome-wide association study
PRS: Polygenic risk score
