# Supplementary material for: Prediction of esophageal cancer risk based on genetic variants and environmental risk factors in Chinese population
Source: BMC Cancer. 2024 May 16;24:598. doi: 10.1186/s12885-024-12370-y (PMC11100074; doi:10.1186/s12885-024-12370-y)
Supplement: Supplementary file 1 — Additional file 1: Figure S1. Flow chart of literature selection in the meta-analysis. Figure S2. Distribution of studies included in mete-analysis by province in China. (docx) [file 12885_2024_12370_MOESM1_ESM.docx]

**Supplementary Figures**


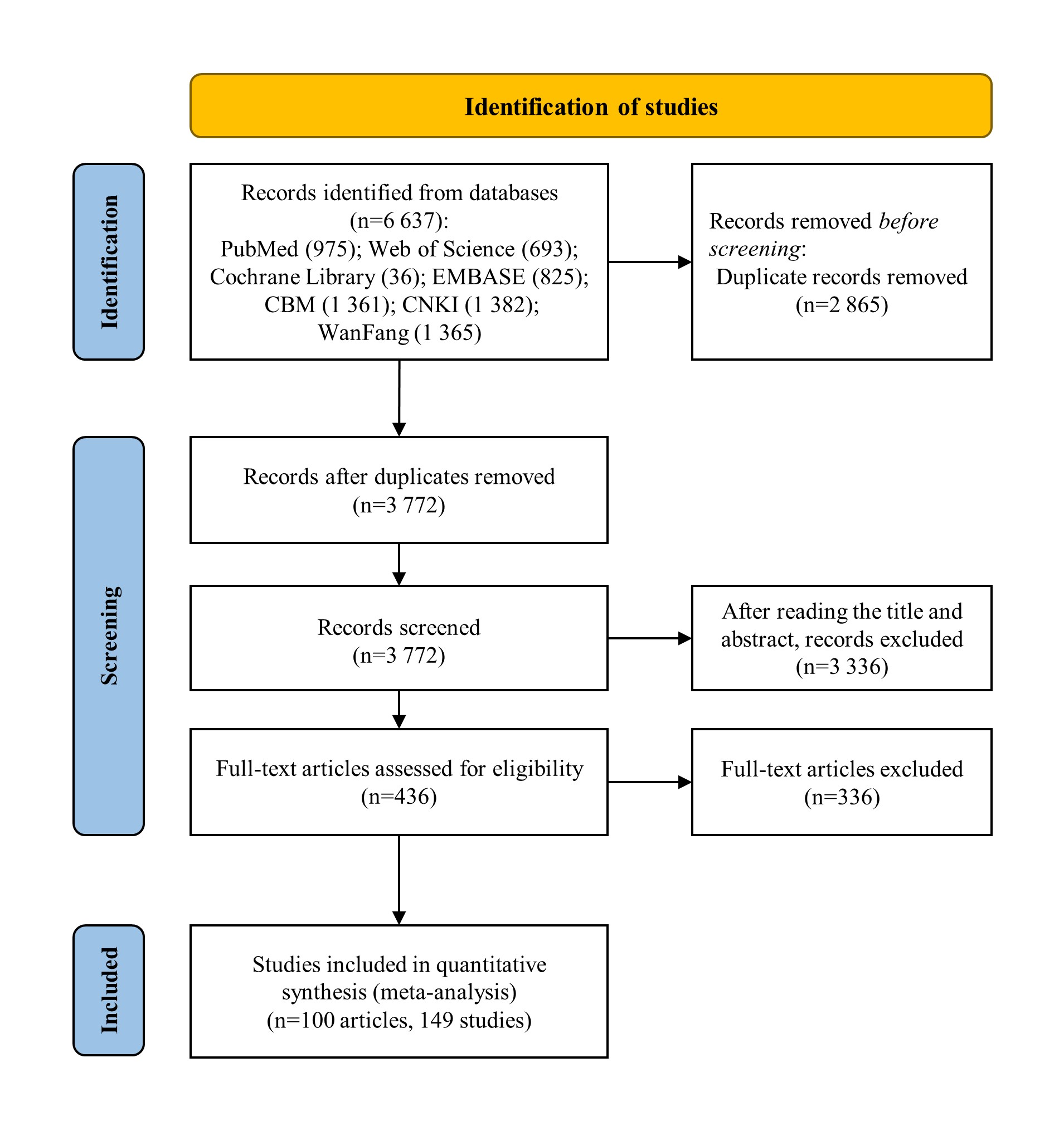


**Figure S1.** Flow chart of literature selection in the meta-analysis.


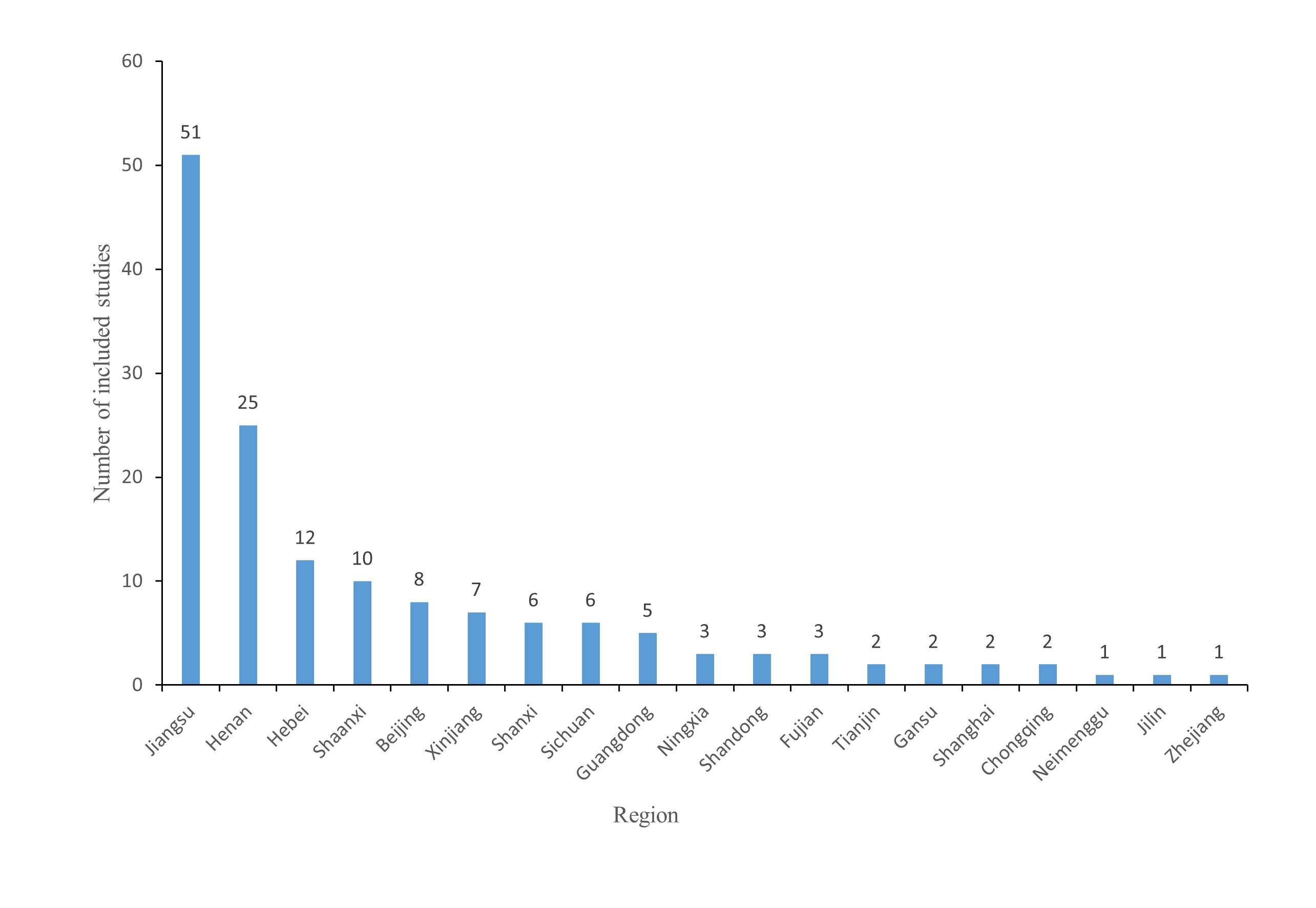
**Figure S2.** Distribution of studies included in mete-analysis by province in China.
