# Supplementary material for: Prediction of esophageal cancer risk based on genetic variants and environmental risk factors in Chinese population
Source: BMC Cancer. 2024 May 16;24:598. doi: 10.1186/s12885-024-12370-y (PMC11100074; doi:10.1186/s12885-024-12370-y)
Supplement: Supplementary file 2 — Additional file 2: Table S1. SNPs identified from the meta-analysis. Table S2. Associations of genetic variants with esophageal cancer risk in the meta-analysis. Table S3. Heterogeneity test and evaluation of reliability for genetic variants significantly associated with esophageal cancer risk. Table S4. Epidemiological effect estimation for the relationship between genetic variants and esophageal cancer. Table S5. Associations of 14 candidate SNPs with risk of esophageal cancer in the case-control study. Table S6. Associations of genotypes of 14 candidate SNPs with esophageal cancer risk. Table S7. False positive report probability of 5 promising SNPs. Table S8. Risk score for each promising SNP. Table S9. Construction of non-genetic and combined models. (docx) [file 12885_2024_12370_MOESM2_ESM.docx]

**Supplementary Tables**

| **Table S1.** SNPs identified from the meta-analysis. | | | | | | | | | |
| --- | --- | --- | --- | --- | --- | --- | --- | --- | --- |
| Gene | SNP | Chr | Position | Risk allele | Compliance with HWE | NOS≥7 | Source of control | Quality control | Cases/controls |
|  |  |  |  |  | (n) | (n) | (PB/HB) | (n) |  |
| *P53* | rs1042522 | 17 | 7579472 | C | 7 | 5 | 6/1 | 4 | 2182/2484 |
| *CYP1A1* | rs1048943 | 15 | 75012985 | C | 8 | 6 | 4/4 | 1 | 1185/1166 |
|  | rs4646903 | 15 | 75011641 | T | 6 | 5 | 2/4 | 1 | 761/813 |
| *ADH1B* | rs1229984 | 4 | 100239319 | C | 9 | 6 | 8/1 | 4 | 5076/6291 |
| *ERCC2* | rs13181 | 19 | 45854919 | G | 10 | 5 | 9/1 | 3 | 3008/3532 |
|  | rs1799793 | 19 | 45867259 | A | 4 | 2 | 4/0 | 2 | 1457/1800 |
| *XRCC1* | rs1799782 | 19 | 44057574 | C | 5 | 4 | 3/2 | 3 | 1296/2013 |
|  | rs25487 | 19 | 44055726 | A | 6 | 4 | 5/1 | 3 | 1543/2403 |
| *NQO1* | rs1800566 | 16 | 69745145 | A | 6 | 5 | 2/4 | 3 | 1426/1529 |
| *XPA* | rs1800975 | 9 | 100459578 | G | 3 | 3 | 2/1 | 2 | 673/1215 |
| *MTHFR* | rs1801133 | 1 | 11856378 | A | 8 | 7 | 5/3 | 6 | 1689/2745 |
| *RAD51* | rs1801320 | 15 | 40987528 | C | 3 | 0 | 2/1 | 1 | 537/457 |
| *IL23R* | rs1884444 | 1 | 67633812 | G | 4 | 4 | 3/1 | 3 | 3880/4312 |
|  | rs6682925 | 1 | 67631262 | T | 3 | 3 | 3/0 | 3 | 3374/3805 |
| *CYP2E1* | rs2031920 | 10 | 135339845 | T | 6 | 4 | 5/1 | 0 | 679/785 |
|  | rs3813867 | 10 | 135339605 | C | 3 | 2 | 3/0 | 1 | 768/815 |
|  | rs6413432 | 10 | 135348544 | T | 3 | 2 | 1/2 | 0 | 261/303 |
| *MMP13* | rs2252070 | 11 | 102826539 | G | 3 | 3 | 3/0 | 3 | 1904/2209 |
| *PLCE1* | rs2274223 | 10 | 96066341 | G | 7 | 3 | 6/1 | 3 | 3269/3458 |
| *CDKN1A* | rs1059234 | 6 | 36653597 | T | 3 | 3 | 2/1 | 3 | 1130/1388 |
|  | rs2395655 | 6 | 36645696 | G | 3 | 3 | 2/1 | 2 | 1384/1445 |
| *MDM2* | rs150550023 | 12 | 69200806 | Ins | 3 | 1 | 2/1 | 3 | 709/1000 |
| *MMP9* | rs3918242 | 20 | 44635976 | T | 3 | 1 | 3/0 | 1 | 584/718 |
| *TERT* | rs401681 | 5 | 1322087 | T | 3 | 1 | 2/1 | 1 | 1489/1596 |
| *ALDH2* | rs671 | 12 | 112241766 | A | 11 | 6 | 10/1 | 4 | 4518/5713 |
| *PTEN* | rs701848 | 10 | 89726745 | C | 3 | 3 | 3/0 | 2 | 1040/1193 |
| *FasL* | rs763110 | 1 | 172627498 | C | 3 | 2 | 3/0 | 2 | 1040/1193 |
| *GSTM1* | *-* | 1 | 109694069 | Null | 8 | 6 | 4/4 | 0 | 984/1089 |
| *GSTT1* | - | 22 | 270452 | Null | 5 | 3 | 3/2 | 0 | 643/765 |
| NOTE: SNP, single nucleotide polymorphism; Chr, chromosome; HWE, Hardy-Weinberg equilibrium; PB, population-based; HB, hospital-based. | | | | | | | | | |

| **Table S2.** Associations of genetic variants with esophageal cancer risk in the meta-analysis. | | | | | |
| --- | --- | --- | --- | --- | --- |
| Gene rs# | Per-allele | Heterozygous | Homozygous | Recessive model | Dominant model |
|  | OR (95%CI) | OR (95%CI) | OR (95%CI) | OR (95%CI) | OR (95%CI) |
| *P53* rs1042522 | **1.21 (1.02,1.44)** | **1.19 (1.04,1.37)** | **1.42 (1.02,1.99)** | 1.24 (0.90,1.69) | **1.25 (1.10,1.43)** |
| *CYP1A1* rs1048943 | **1.52 (1.33,1.73)** | **1.62 (1.35,1.94)** | **2.44 (1.79,3.33)** | **2.06 (1.57,2.71)** | **1.74 (1.46,2.07)** |
| *ADH1B* rs1229984 | **1.39 (1.31,1.47)** | **1.20 (1.11,1.30)** | **2.23 (1.78,2.79)** | **2.05 (1.63,2.58)** | **1.36 (1.26,1.46)** |
| *ERCC2* rs13181 | **1.25 (1.08,1.46)** | **1.25 (1.11,1.41)** | **1.77 (1.30,2.41)** | **1.61(1.18,2.19)** | **1.24 (1.04,1.47)** |
| *XRCC1* rs1799782 | 1.08 (0.97,1.21) | 0.99 (0.85,1.15) | 1.19 (0.93,1.53) | 1.20 (0.94,1.52) | 1.02 (0.89,1.18) |
| *ERCC2* rs1799793 | 1.11 (0.91,1.35) | 1.10 (0.89,1.35) | 1.51 (0.48,4.73) | 1.49 (0.48,4.67) | 1.11 (0.90,1.36) |
| *NQO1* rs1800566 | 1.33 (0.99,1.78) | **1.45 (1.01,2.09)** | 1.82 (0.96,3.44) | 1.39 (0.88,2.21) | **1.56 (1.02,2.39)** |
| *XPA* rs1800975 | 1.15 (0.67,1.99) | 0.99 (0.56,1.74) | 1.26 (0.46,3.47) | 1.28 (0.62,2.62) | 1.09 (0.52,2.30) |
| *MTHFR* rs1801133 | 1.29 (0.92,1.79) | 1.32 (0.83,2.09) | 1.18 (0.52,2.68) | 1.08 (0.62,1.85) | 1.29 (0.75,2.22) |
| *RAD51* rs1801320 | 1.42 (0.88,2.31) | 1.41 (0.75,2.64) | 1.66 (0.82,3.35) | 1.59 (0.79,3.19) | 1.47 (0.81,2.66) |
| *IL23R* rs1884444 | 1.01 (0.87,1.18) | 1.05 (0.91,1.21) | 1.05 (0.79,1.39) | 0.98 (0.83,1.17) | 1.01 (0.88,1.16) |
| *CYP2E1* rs2031920 | 0.76 (0.53,1.09) | 0.79 (0.54,1.16) | 0.62 (0.30,1.29) | 0.68 (0.36,1.27) | 0.73 (0.49,1.07) |
| *MMP13* rs2252070 | **0.80 (0.65,0.98)** | **0.73 (0.56,0.95)** | **0.65 (0.45,0.96)** | **0.78 (0.62,0.98)** | **0.71 (0.53,0.95)** |
| *PLCE1* rs2274223 | **1.39 (1.28,1.51)** | **1.38 (1.25,1.53)** | **1.96 (1.59,2.42)** | **1.73 (1.40,2.13)** | **1.45 (1.31,1.60)** |
| *CDKN1A* rs1059234 | 1.08 (0.84,1.39) | 0.80 (0.46,1.37) | 0.67 (0.24,1.90) | 0.80 (0.41,1.57) | 0.74 (0.38,1.46) |
| *CDKN1A* rs2395655 | 1.09 (0.90,1.32) | **1.40 (1.16,1.70)** | 1.23 (0.86,1.77) | 0.96 (0.67,1.38) | **1.33 (1.11,1.59)** |
| *XRCC1* rs25487 | 1.05 (0.88,1.25) | 0.95 (0.83,1.09) | 1.39 (0.90,2.17) | 1.44 (0.93,2.22) | 1.01 (0.89,1.15) |
| *MDM2* rs150550023 | 1.13 (0.97,1.31) | 1.22 (0.87,1.70) | 1.23 (0.84,1.79) | 1.12 (0.91,1.38) | 1.27 (0.92,1.74) |
| *CYP2E1* rs3813867 | **0.63 (0.53,0.75)** | **0.65 (0.52,0.80)** | **0.34 (0.20,0.59)** | **0.40 (0.23,0.68)** | **0.61 (0.49,0.75)** |
| *MMP9* rs3918242 | 1.08 (0.69,1.71) | 1.10 (0.80,1.50) | 1.34 (0.26,6.96) | 0.94 (0.64,1.40) | 0.97 (0.51,1.84) |
| *TERT* rs401681 | **0.80 (0.72,0.90)** | **0.68 (0.58,0.79)** | **0.78 (0.61,0.98)** | 0.93 (0.74,1.16) | **0.70 (0.60,0.81)** |
| *CYP1A1* rs4646903 | 1.27 (0.94,1.71) | **1.40 (1.13,1.74)** | 1.37 (0.70,2.67) | 1.15 (0.65,2.05) | **1.43 (1.02,1.99)** |
| *CYP2E1* rs6413432 | 1.01 (0.78,1.32) | 0.99 (0.48,2.04) | 1.02 (0.50,2.07) | 1.01 (0.73,1.42) | 1.01 (0.51,2.02) |
| *IL23R* rs6682925 | 0.94 (0.85,1.04) | 1.05 (0.91,1.20) | 0.91 (0.79,1.04) | **0.87 (0.79,0.96)** | 0.98 (0.86,1.12) |
| *ALDH2* rs671 | 1.16 (0.88,1.52) | 1.17 (0.96,1.43) | 0.78 (0.45,1.37) | 0.73 (0.42,1.27) | 1.14 (0.94,1.39) |
| *PTEN* rs701848 | 1.13 (0.80,1.60) | 1.22 (0.77,1.92) | 1.37 (0.60,2.71) | 1.13 (0.65,1.99) | 1.21 (0.75,1.96) |
| *FASL* rs763110 | 1.03 (0.56,1.90) | 0.95 (0.43,2.09) | 2.71 (0.42,2.87) | 1.12 (0.61,2.08) | 1.01 (0.44,2.28) |
| *GSTM1*^a^ | 1.11 (0.76,1.64) | - | - | - | - |
| *GSTT1*^a^ | 1.15 (0.93,1.44) | - | - | - | - |
| NOTE: A, wild-type allele; B, mutant allele.  ^a^ Null versus normal. | | | |  |  |

| **Table S3.** Heterogeneity test and evaluation of reliability for genetic variants significantly associated with esophageal cancer risk. | | | | | | | | | | | |
| --- | --- | --- | --- | --- | --- | --- | --- | --- | --- | --- | --- |
| Gene | SNP | Genetic model | Heterogeneity test | | | Model^a^ | OR (95%CI) | *P* value | FPRP | FPRP | FPRP |
|  |  |  | Q | *I^2^* | *P* value |  |  |  | (0.25) | (0.1) | (0.01) |
| *P53* | rs1042522 | G vs C | 19.81 | 69.70% | 0.003 | R | 1.21(1.02,1.44) | 0.029 | **0.081** | **0.209** | 0.744 |
| *P53* | rs1042522 | GG vs GC+CC | 10.57 | 43.20% | 0.103 | F | 1.25 (1.10,1.43) | 0.001 | **0.002** | **0.007** | **0.075** |
| *CYP1A1* | rs1048943 | T vs C | 8.36 | 16.30% | 0.302 | F | 1.52 (1.33,1.73) | <0.001 | **<0.001** | **<0.001** | **<0.001** |
| *CYP1A1* | rs1048943 | TT vs CC | 13.20 | 47.00% | 0.067 | F | 2.44 (1.79,3.33) | <0.001 | **<0.001** | **<0.001** | **0.002** |
| *ADH1B* | rs1229984 | T vs C | 15.20 | 47.40% | 0.055 | F | 1.39 (1.31,1.47) | <0.001 | **<0.001** | **<0.001** | **<0.001** |
| *ADH1B* | rs1229984 | TT vs TC | 2.21 | 0.00% | 0.974 | F | 1.20 (1.11,1.30) | <0.001 | **<0.001** | **<0.001** | **0.001** |
| *ERCC2* | rs13181 | T vs G | 17.61 | 48.90% | 0.040 | R | 1.25 (1.08,1.46) | 0.004 | **0.012** | **0.034** | **0.277** |
| *ERCC2* | rs13181 | TT vs TG+GG | 17.78 | 49.40% | 0.038 | R | 1.24 (1.04,1.47) | 0.015 | **0.045** | **0.125** | 0.611 |
| *NQO1* | rs1800566 | GG vs GA | 16.02 | 68.80% | 0.007 | R | 1.45 (1.01,2.09) | 0.047 | **0.198** | **0.425** | 0.891 |
| *NQO1* | rs1800566 | GG vs GA+AA | 24.72 | 79.80% | <0.001 | R | 1.56 (1.02,2.39) | 0.040 | **0.218** | **0.455** | 0.902 |
| *MMP13* | rs2252070 | C vs T | 9.73 | 79.40% | 0.008 | R | 0.80 (0.65,0.98) | 0.030 | **0.084** | **0.216** | 0.752 |
| *MMP13* | rs2252070 | CC vs CT+TT | 8.54 | 76.60% | 0.014 | R | 0.71 (0.53,0.95) | 0.020 | **0.085** | **0.219** | 0.755 |
| *PLCE1* | rs2274223 | A vs G | 9.10 | 34.10% | 0.168 | F | 1.39 (1.28,1.51) | <0.001 | **<0.001** | **<0.001** | **0.001** |
| *PLCE1* | rs2274223 | AA vs GG | 7.51 | 20.10% | 0.276 | F | 1.96 (1.59,2.42) | <0.001 | **<0.001** | **<0.001** | **0.001** |
| *CDKN1A* | rs2395655 | AA vs AG | 1.99 | 0.00% | 0.370 | F | 1.40 (1.16,1.70) | <0.001 | **0.002** | **0.005** | **0.056** |
| *CDKN1A* | rs2395655 | AA vs AG+GG | 2.60 | 23.00% | 0.273 | F | 1.33 (1.11,1.59) | 0.002 | **0.007** | **0.021** | **0.190** |
| *CYP2E1* | rs3813867 | G vs C | 0.08 | 0.00% | 0.960 | F | 0.63 (0.53,0.75) | <0.001 | **<0.001** | **<0.001** | **<0.001** |
| *CYP2E1* | rs3813867 | GG vs CC | 0.67 | 0.00% | 0.716 | F | 0.34 (0.20,0.59) | <0.001 | **0.039** | **0.110** | 0.575 |
| *CYP2E1* | rs3813867 | GG+GC vs CC | 0.70 | 0.00% | 0.704 | F | 0.40 (0.23,0.68) | <0.001 | **0.072** | **0.188** | 0.718 |
| *TERT* | rs401681 | C vs T | 0.05 | 0.00% | 0.978 | F | 0.80 (0.72,0.90) | <0.001 | **<0.001** | **0.001** | **0.009** |
| *TERT* | rs401681 | CC vs TT | 0.18 | 0.00% | 0.912 | F | 0.78 (0.61,0.98) | 0.035 | **0.106** | **0.263** | 0.797 |
| *CYP1A1* | rs4646903 | AA vs AG | 6.83 | 26.80% | 0.233 | F | 1.40 (1.13,1.74) | 0.002 | **0.010** | **0.029** | **0.246** |
| *CYP1A1* | rs4646903 | AA vs AG+GG | 12.19 | 59.00% | 0.032 | R | 1.43 (1.02,1.99) | 0.037 | **0.153** | **0.351** | 0.856 |
| *IL23R* | rs6682925 | CC+CT vs TT | 2.29 | 12.50% | 0.319 | F | 0.87 (0.79,0.96) | 0.007 | **0.020** | **0.058** | **0.406** |
| NOTE: FPRP, false positive report probability (at three levels of prior probability, see text for more details).  ^a^ Model: F, fixed-effects model; R, random-effects model. | | | | | | | | | | |  |

| **Tabel S4.** Epidemiological effect estimation for the relationship between genetic variants and esophageal cancer. | | | | | | | | | |
| --- | --- | --- | --- | --- | --- | --- | --- | --- | --- |
| Gene | SNP | Chr | Genetic model | OR (95%CI) | Risk allele frequency | | Epidemiological effect evaluation | | |
|  |  |  |  |  | Control | CHB | *ARP* (%) | *PARP*^Control^ (%) | *PARP*^CHB^ (%) |
| *P53* | rs1042522 | 17 | GG vs CC | 1.42(1.02,1.99) | 0.461 | 0.452 | 29.58 | 16.22 | 15.96 |
| *CYP1A1* | rs1048943 | 15 | TT vs CC | 2.44(1.79,3.33) | 0.276 | 0.267 | 59.02 | 28.44 | 27.77 |
| *ADH1B* | rs1229984 | 4 | TT vs CC | 2.23(1.78,2.79) | 0.291 | 0.291 | 55.16 | 26.36 | 26.36 |
| *ERCC2* | rs13181 | 19 | TT vs GG | 1.77(1.30,2.41) | 0.123 | 0.112 | 43.5 | 8.65 | 7.94 |
| *NQO1* | rs1800566 | 16 | CC vs CT+TT | 1.56(1.02,2.39) | 0.441 | 0.5 | 35.9 | 19.8 | 21.88 |
| *MMP13* | rs2252070* | 11 | TT vs CC | 1.53(1.04,2.24) | 0.525 | 0.515 | 34.64 | 21.77 | 21.44 |
| *PLCE1* | rs2274223 | 10 | AA vs GG | 1.96(1.59,2.42) | 0.213 | 0.189 | 48.98 | 16.98 | 15.36 |
| *CDKN1A* | rs2395655 | 6 | AA vs AG | 1.40(1.16,1.70) | 0.538 | 0.515 | 28.57 | 17.71 | 17.08 |
| *CYP2E1* | rs3813867* | 10 | CC vs GG | 2.93(1.70,5.05) | 0.757 | 0.762 | 65.87 | 59.37 | 59.52 |
| *TERT* | rs401681* | 5 | CT vs CC | 1.48(1.26,1.72) | 0.65 | 0.723 | 32.43 | 23.78 | 25.76 |
| *CYP1A1* | rs4646903 | 15 | AA vs AG+GG | 1.43(1.02,1.99) | 0.358 | 0.437 | 30.07 | 13.34 | 15.82 |
| *IL23R* | rs6682925* | 1 | TT vs TC+CC | 1.14(1.04,1.26) | 0.384 | 0.515 | 12.28 | 5.1 | 6.73 |
| NOTE: The 12 significant SNPs were identified by the use of meta-analysis. Chr, chromosome; CHB, Chinese Han in Beijing, China; *ARP*, attributable risk percentage; *PARP*, population attribute risk percentage.  *With mutant genotypes as a reference.  *ARP* = \|*OR*-1/*OR*\|×100%; *PARP* = \| *P*_e_(*OR*-1)/[*P*_e_(*OR*-1)+1] \| ×100%, *P*_e_ was the risk allele frequency in the controls or general population; The risk gene frequencies of both the control group and CHB in the 1000 Genomes Browser were used as exposure rate when calculating *PARP*. | | | | | | | | | |

| **Table S5.** Associations of 14 candidate SNPs with risk of esophageal cancer in the case-control study. | | | | | | | | |
| --- | --- | --- | --- | --- | --- | --- | --- | --- |
| SNP | Allele model | | Recessive model | | Dominant model | | Super dominant model | |
|  | OR (95%CI) | *P* value | OR (95%CI) | *P* value | OR (95%CI) | *P* value | OR (95%CI) | *P* value |
| rs1042522 | **0.80(0.67,0.96)** | **0.015** | **0.69(0.48,1.00)*** | **0.047** | 0.84(0.55,1.26) | 0.388 | 1.21(0.87,1.69) | 0.255 |
| rs1048943 | 1.09(0.88,1.35) | 0.418 | 1.02(0.46,2.28) | 0.953 | 1.33(0.95,1.88)* | 0.099 | 1.35(0.95,1.92) | 0.096 |
| rs1229984 | 1.15(0.96,1.39) | 0.132 | **1.78(1.08,2.94)*** | **0.024** | 1.20(0.86,1.68) | 0.282 | 0.93(0.66,1.29) | 0.649 |
| rs13181 | 0.95(0.69,1.31) | 0.743 | 1.07(0.16,7.23) | 0.942 | 0.87(0.56,1.36)* | 0.544 | 0.86(0.55,1.36) | 0.525 |
| rs1800566 | 1.00(0.84,1.20) | 0.964 | 1.08(0.71,1.65) | 0.711 | 1.10(0.76,1.59)* | 0.611 | 1.03(0.74,1.44) | 0.866 |
| rs1801133 | **0.75(0.62,0.89)** | **0.001** | 0.71(0.50,1.01) | 0.054 | **0.41(0.26,0.66)*** | **<0.001** | 0.85(0.61,1.18) | 0.326 |
| rs2252070 | 0.94(0.79,1.12) | 0.474 | 0.94(0.63,1.39) | 0.742 | 0.99(0.68,1.45) | 0.964 | 1.04(0.75,1.45)* | 0.813 |
| rs2274223 | **1.68(1.37,2.07)** | **<0.001** | **2.32(1.22,4.42)** | **0.011** | **1.93(1.37,2.71)*** | **<0.001** | **1.56(1.09,2.23)** | **0.014** |
| rs2395655 | 0.93(0.78,1.11) | 0.419 | 0.87(0.61,1.26)* | 0.471 | 0.94(0.62,1.41) | 0.758 | 1.07(0.77,1.49) | 0.685 |
| rs3813867 | 0.89(0.72,1.11) | 0.312 | 0.78(0.31,1.98) | 0.601 | 1.14(0.81,1.61) | 0.462 | 1.19(0.83,1.69)* | 0.341 |
| rs401681 | 0.89(0.74,1.07) | 0.202 | 0.75(0.45,1.26)* | 0.275 | 0.95(0.68,1.33) | 0.953 | 1.08(0.77,1.50) | 0.667 |
| rs4646903 | 1.11(0.93,1.34) | 0.247 | 1.15(0.72,1.85) | 0.552 | 1.15(0.82,1.61)* | 0.422 | 1.07(0.76,1.49) | 0.709 |
| rs6682925 | 0.98(0.82,1.18) | 0.855 | 1.06(0.75,1.50) | 0.761 | 1.45(0.93,2.26)* | 0.102 | 1.17(0.84,1.63) | 0.353 |
| rs671 | **1.44(1.13,1.85)** | **0.003** | 1.93(0.55,6.77) | 0.307 | **2.42(1.65,3.56)*** | **<0.001** | **2.34(1.59,3.45)** | **<0.001** |
| NOTE: Logistic regression was performed with adjustment for age, smoking, drinking and family history of esophageal cancer.  *The best-fit genetic model for each SNP is determined based on the maximum likelihood ratio. | | | | | | | | |

| **Table S6.** Associations of genotypes of 14 candidate SNPs with esophageal cancer risk. | | | | | | | | |
| --- | --- | --- | --- | --- | --- | --- | --- | --- |
| SNP | Cases (%) | Controls (%) | *P*_HWE_ value^a^ | *P* value^b^ | OR (95%CI) | *P* value^c^ | OR (95%CI) | *P* value^d^ |
|  |  |  |  |  |  |  |  |  |
| rs1042522 |  |  | 0.551 | 0.038 |  |  |  |  |
| GG | 112(22.4) | 94(18.8) |  |  | Reference |  | Reference |  |
| GC | 256(51.2) | 238(47.6) |  |  | 0.90(0.65,1.25) | 0.539 | 0.95(0.61,1.49) | 0.823 |
| CC | 132(26.4) | 168(33.6) |  |  | **0.66(0.46,0.92)** | **0.022** | 0.69(0.43,1.09) | 0.112 |
| rs1048943 |  |  | 0.726 | 0.6 |  |  |  |  |
| TT | 296(59.2) | 311(62.2) |  |  | Reference |  | Reference |  |
| TC | 180(36.0) | 165(33.0) |  |  | 1.15(0.88,1.49) | 0.312 | 1.36(0.95,1.94) | 0.091 |
| CC | 24(4.8) | 24(4.8) |  |  | 1.05(0.58,1.89) | 0.869 | 1.14(0.49,2.65) | 0.764 |
| rs1229984 |  |  | 0.806 | 0.247 |  |  |  |  |
| TT | 212(42.4) | 227(45.4) |  |  | Reference |  | Reference |  |
| TC | 216(43.2) | 218(43.6) |  |  | 1.06(0.81,1.38) | 0.662 | 1.06(0.74,1.52) | 0.735 |
| CC | 72(14.4) | 55(11.0) |  |  | 1.40(0.94,2.09) | 0.096 | **1.78(1.06,3.01)** | **0.031** |
| rs13181 |  |  | 0.794 | 0.939 |  |  |  |  |
| TT | 424(84.0) | 420(84.8) |  |  | Reference |  | Reference |  |
| TG | 73(15.4) | 77(14.6) |  |  | 0.94(0.66,1.33) | 0.723 | 0.86(0.55,1.36) | 0.525 |
| GG | 3(0.6) | 3(0.6) |  |  | 0.99(0.20,4.93) | 0.991 | 1.09(0.16,7.24) | 0.932 |
| rs1800566 |  |  | 0.557 | 0.997 |  |  |  |  |
| GG | 141(28.2) | 142(28.4) |  |  | Reference |  | Reference |  |
| GA | 256(51.2) | 255(51.0) |  |  | 1.01(0.76,1.35) | 0.941 | 1.09(0.74,1.59) | 0.666 |
| AA | 103(20.6) | 103(20.6) |  |  | 1.01(0.70,1.44) | 0.969 | 1.10(0.67,1.82) | 0.707 |
| rs1801133 |  |  | 0.973 | 0.01 |  |  |  |  |
| GG | 100(20.0) | 69(13.8) |  |  | Reference |  | Reference |  |
| GA | 237(47.4) | 233(46.6) |  |  | 0.70(0.49,1.00) | 0.051 | **0.44(0.27,0.72)** | **0.001** |
| AA | 163(32.6) | 198(39.6) |  |  | **0.57(0.39,0.82)** | **0.003** | **0.37(0.22,0.62)** | **<0.001** |
| rs2252070 |  |  | 0.647 | 0.758 |  |  |  |  |
| CC | 135(27.0) | 128(25.6) |  |  | Reference |  | Reference |  |
| CT | 257(51.4) | 255(51.0) |  |  | 0.96(0.71,1.29) | 0.765 | 1.01(0.68,1.51) | 0.946 |
| TT | 108(21.6) | 117(23.4) |  |  | 0.88(0.61,1.25) | 0.463 | 0.96(0.60,1.55) | 0.879 |
| rs2274223 |  |  | 0.369 | <0.001 |  |  |  |  |
| AA | 252(50.4) | 324(64.8) |  |  | Reference |  | Reference |  |
| AG | 201(40.2) | 153(30.6) |  |  | **1.69(1.29,2.21)** | **<0.001** | **1.76(1.22,2.53)** | **0.002** |
| GG | 47(9.4) | 23(4.6) |  |  | **2.63(1.55,4.44)** | **<0.001** | **2.79(1.45,5.38)** | **0.002** |
| rs2395655 |  |  | 0.885 | 0.659 |  |  |  |  |
| AA | 105(21.0) | 100(20.0) |  |  | Reference |  | Reference |  |
| AG | 257(51.4) | 249(49.8) |  |  | 0.98(0.71,1.36) | 0.917 | 0.98(0.63,1.52) | 0.929 |
| GG | 138(27.6) | 151(30.2) |  |  | 0.87(0.61,1.25) | 0.447 | 0.86(0.54,1.38) | 0.534 |
| rs3813867 |  |  | 0.668 | 0.236 |  |  |  |  |
| GG | 324(64.8) | 316(63.2) |  |  | Reference |  | Reference |  |
| GC | 163(32.6) | 161(32.2) |  |  | 0.99(0.76,1.29) | 0.926 | 1.18(0.82,1.68) | 0.378 |
| CC | 13(2.6) | 23(4.6) |  |  | 0.55(0.27,1.11) | 0.094 | 0.84(0.32,2.20) | 0.721 |
|  |  |  |  |  |  |  |  |  |
| **Table S6. (continued)** | | | | | | | | |
| SNP | Cases (%) | Controls (%) | *P*_HWE_ value^a^ | *P* value^b^ | OR (95%CI) | *P* value^c^ | OR (95%CI) | *P* value^d^ |
|  |  |  |  |  |  |  |  |  |
| rs401681 |  |  | 0.993 | 0.299 |  |  |  |  |
| CC | 222(44.4) | 210(42.0) |  |  | Reference |  | Reference |  |
| CT | 231(46.2) | 228(45.6) |  |  | 0.96(1.74,1.25) | 0.751 | 1.01(0.71,1.44) | 0.94 |
| TT | 47(9.4) | 62(12.4) |  |  | 0.72(0.47,1.10) | 0.124 | 0.77(0.45,1.31) | 0.332 |
| rs4646903 |  |  | 0.376 | 0.513 |  |  |  |  |
| AA | 193(38.6) | 210(42.0) |  |  | Reference |  | Reference |  |
| AG | 230(46.0) | 221(44.2) |  |  | 1.13(0.87,1.48) | 0.365 | 1.13(0.79,1.62) | 0.506 |
| GG | 77(15.4) | 69(13.8) |  |  | 1.21(0.83,1.77) | 0.316 | 1.23(0.74,2.04) | 0.431 |
| rs6682925 |  |  | 0.739 | 0.927 |  |  |  |  |
| CC | 82(16.4) | 83(16.6) |  |  | Reference |  | Reference |  |
| CT | 243(48.6) | 237(47.4) |  |  | 1.04(0.73,1.48) | 0.837 | 1.46(0.91,2.33) | 0.119 |
| TT | 175(35.0) | 180(36.0) |  |  | 0.98(0.68,1.42) | 0.932 | 1.39(0.85,2.27) | 0.194 |
| rs671 |  |  | 0.746 | 0.007 |  |  |  |  |
| GG | 336(67.2) | 381(76.2) |  |  | Reference |  | Reference |  |
| GA | 153(30.6) | 110(22.0) |  |  | **1.58(1.19,2.10)** | **0.002** | **2.41(1.63,3.56)** | **<0.001** |
| AA | 11(2.2) | 9(1.8) |  |  | 1.39(0.57,3.39) | 0.474 | 2.405(0.67,8.66) | 0.18 |
| NOTE: SNP, single nucleotide polymorphism; HWE, Hardy-Weinberg equilibrium. | | | | | | | | |
| ^a^*P* value of HWE in controls. | | | | | | | | |
| ^b^Differences in genotype distribution between case and control groups were compared using χ2 test. | | | | | | | | |
| ^c^Logistic regression was used without any adjustment. | | | | | | | | |
| ^d^Logistic regression was used with adjustment for age, smoking, alcohol consumption and family history of esophageal cancer. | | | | | | | | |

| **Table S7.** False positive report probability of 5 promising SNPs. | | | | | |
| --- | --- | --- | --- | --- | --- |
| SNP | OR (95%CI) | *P* value | FPRP | | |
|  |  |  | (0.25) | (0.10) | (0.01) |
| rs1042522 recessive | 0.69(0.48,1.00) | 0.047 | **0.193** | **0.418** | 0.888 |
| rs1229984 recessive | 1.78(1.08,2.94) | 0.024 | **0.225** | **0.466** | 0.906 |
| rs1801133 dominant | 0.41(0.26,0.66) | <0.001 | **0.027** | **0.076** | **0.474** |
| rs2274223 dominant | 1.93(1.37,2.71) | <0.001 | **0.007** | **0.021** | **0.189** |
| rs671 dominant | 2.42(1.65,3.56) | <0.001 | **0.003** | **0.008** | **0.081** |
| NOTE: FPRP, false positive report probability. | | | | | |

| **Table S8.** Risk score for each promising SNP. | | | | |
| --- | --- | --- | --- | --- |
| SNP | Risk allele | Risk allele frequency | OR | Risk score |
|  |  |  |  |  |
| rs1042522 | G | 0.548 | 1.24 |  |
| CC |  |  |  | 0.778 |
| CG |  |  |  | 0.968 |
| GG |  |  |  | 1.204 |
| rs1229984 | C | 0.291 | 1.15 |  |
| TT |  |  |  | 0.917 |
| TC |  |  |  | 1.057 |
| CC |  |  |  | 1.218 |
| rs1801133 | G | 0.534 | 1.32 |  |
| AA |  |  |  | 0.732 |
| AG |  |  |  | 0.963 |
| GG |  |  |  | 1.268 |
| rs2274223 | G | 0.189 | 1.68 |  |
| AA |  |  |  | 0.784 |
| AG |  |  |  | 1.321 |
| GG |  |  |  | 2.224 |
| rs671 | A | 0.160 | 1.45 |  |
| GG |  |  |  | 0.871 |
| GA |  |  |  | 1.259 |
| AA |  |  |  | 1.820 |
| Note: Promising SNPs verified in the case-control study were utilized to calculate the weighted genetic risk score (wGRS). OR was the OR of the risk allele. | | | | |

| **Table S9.** Construction of non-genetic and combined models. | | | | |
| --- | --- | --- | --- | --- |
| Risk factors | β^a^ | OR | 95%CI | *P* value |
| **Non-genetic model** |  |  |  |  |
| Smoking (X_1_) | -0.584 | 0.56 | (0.34,0.92) | 0.022 |
| No (0) |  |  |  |  |
| Yes (1) |  |  |  |  |
| Family history of esophageal cancer (X_2_) | 2.038 | 7.67 | (3.17,18.55) | <0.001 |
| No (0) |  |  |  |  |
| Yes (1) |  |  |  |  |
| Smoking*alcohol (X_3_) | 1.392 | 4.02 | (2.27,7.13) | <0.001 |
| Constant | -0.236 | 0.79 | - | 0.03 |
| **Combined model** |  |  |  |  |
| wGRS (X_1_) | 0.908 | 2.48 | (1.70,3.62) | <0.001 |
| Smoking (X_2_) | -0.558 | 0.57 | (0.34,0.95) | 0.032 |
| No (0) |  |  |  |  |
| Yes (1) |  |  |  |  |
| Family history of esophageal cancer (X_3_) | 1.976 | 7.22 | (2.94,17.73) | <0.001 |
| No (0) |  |  |  |  |
| Yes (1) |  |  |  |  |
| Smoking*alcohol (X_4_) | 1.393 | 4.03 | (2.25,7.20) | <0.001 |
| Constant | -1.110 | 0.33 | - | <0.001 |
| NOTE: Through multivariate logistic regression, the significant variables were further evaluated in the risk prediction model. In the construction of non-genetic model, smoking, alcohol consumption, family history of esophageal cancer, and the interaction between any two of the three factors were analyzed. In the combined model, smoking, alcohol consumption, family history of esophageal cancer, wGRS and the interaction of the four factors were analyzed. wGRS, weighted genetic risk score.  ^a^Regression coefficient. | | | | |
