# Supplementary material for: Prediction of esophageal cancer risk based on genetic variants and environmental risk factors in Chinese population
Source: BMC Cancer. 2024 May 16;24:598. doi: 10.1186/s12885-024-12370-y (PMC11100074; doi:10.1186/s12885-024-12370-y)
Supplement: Supplementary file 3 — Additional file 3: Supplementary References. (docx). [file 12885_2024_12370_MOESM3_ESM.docx]

**Supplementary references**

1. Cao YY, Ge H, Chen LQ, Chen ZF, Wen DG, Li Y, Zhang JH: **Correlation of 53BP1 and p53 Polymorphisms to Susceptibility to Esophageal Squamous Cell Carcinoma and Gastric Cardiac Adenocarcinoma**. *Chinese Journal of Cancer* 2007, **26**(10).

2. Peixoto Guimaraes D, Hsin Lu S, Snijders P, Wilmotte R, Herrero R, Lenoir G, Montesano R, Meijer CJ, Walboomers J, Hainaut P: **Absence of association between HPV DNA, TP53 codon 72 polymorphism, and risk of oesophageal cancer in a high-risk area of China**. *Cancer Lett* 2001, **162**(2):231-235.

3. Zhang JH, Li Y, Wang R, Wen DG, Wu ML, He M: **p53 gene polymorphism with susceptibility to esophageal cancer and lung cancer in Chinese population**. *Chinese Journal of Oncology* 2003, **25**(04):365-367.

4. Li M, Wang D, Wang Y, Sun G, Song W, Zhang B, Borjigin B: **Association of TP53 codon 72 genotype polymorphism and environmental factors with esophageal squamous cell carcinoma in the Mongolian population of the Chinese region of Inner Mongolia**. *Oncol Lett* 2017, **14**(2):1484-1490.

5. Zheng L, Tang WF, Shi YJ, Chen SC, Wang X, Wang LM, Shao AZ, Ding GW, Liu C, Liu RP *et al*: **p21 rs3176352 G > C and p73 rs1801173 C > T Polymorphisms Are Associated with an Increased Risk of Esophageal Cancer in a Chinese Population**. *Plos One* 2014, **9**(5).

6. Shao Y, Tan W, Zhang S: **P53 gene codon 72 polymorphism and risk of esophageal squamous cell carcinoma: a case/control study in a Chinese population**. *Dis Esophagus* 2008, **21**(2):139-143.

7. Ma J, Zhang J, Ning T, Chen Z, Xu C: **Association of genetic polymorphisms in MDM2, PTEN and P53 with risk of esophageal squamous cell carcinoma**. *Journal of Human Genetics* 2012, **57**(4):261-264.

8. Deng YC, Zhang C, Chen Y, Zhang HX, Ma YQ, Ju LT: **Association of esophageal cancer susceptibility with CYP1A1 gene polymorphisms and environmental factors**. *Chinese Journal of Experimental Surgery* 2009, **26**(12):1749-1750.

9. Han YB: **Case-control study of the polymorphisms of the CYP1A1 and GSTM1 genes and susceptibility to esophageal carcinoma**. *Master.* Shanxi Medical University; 2003.

10. Li WY: **A Study of CYP1A1 Gene Polymorphism and the Genetic Susceptibility to Esophageal Carcinoma**. *Master.* Shanxi Medical University; 2002.

11. Wang YP: **CYP1A1 Gene Polymorphisms and Susceptibility to Developing Esophageal Cancer**. *Master.* Zhengzhou University; 2009.

12. Yang J, Yang RS, Li HQ, Wen PE, Jin SK: **Study of a CYP1A1 genotype associated with susceptibility to esophageal cancer**. *Chinese Journal of Clinical Oncology and Rehabilitation* 2004(01):15-17.

13. Wang D, Su M, Tian D, Liang S, Zhang J: **Associations between CYP1A1 and CYP2E1 polymorphisms and susceptibility to esophageal cancer in Chaoshan and Taihang areas of China**. *Cancer Epidemiol* 2012, **36**(3):276-282.

14. Wang AH: **Genetic susceptibility and environment risk markers in the course of esophageal cancer in Xi'an area**. *D.* Air Force Medical University; 2001.

15. Gao P, Tian Y, Ye XF, Ge J, Zhang D, Xu WD: **Study of CTPIA1, GSTT1, GSTM1 polymorphisms and susceptibility on esophageal carcinoma in Ningxia Hui nationality**. *Ningxia Medical Journal* 2012, **34**(3):196-199.

16. Song YH: **Study on the interaction between genetic polymorphisms and environmental risk factors to esophageal cancer in Huai'an population**. *Master.* Southeast University; 2004.

17. Zhang WC: **Effects of Genetic and Environmental Risk Factors to Esophageal Cancer in Huaian Population**. *Master.* Southeast University; 2006.

18. Zhu SJ: **Relationship of the Polymorphisms of CYP1A1 and SULT1A1 Genes and Susceptibility to Esophageal Carcinoma**. *Master.* Shanxi Medical University; 2005.

19. Chen HG: **A match case-control study on esophageal cancer susceptibility with lifestyle habits and genetic polymorphisms of ALDH2 and ADH2**. *Master.* Sichuan University; 2005.

20. Ding JH, Li SP, Cao HX, Wu JZ, Gao CM, Liu YT, Zhou JN, Chang J, Yao GH, Zhou YC: **Relationship of ADH2, ALDH2 Genotypes and Achohol Drinking with Risk of Esophageal Cancer**. *China Cancer* 2010, **19**(7):453-457.

21. Ye B: **Genetic Variant of Single Nucleotide Polymorphism (SNP) is Associated with Risk of Esophageal Squamous Cell Carcinoma**. *PhD.* Peking Union Medical College; 2010.

22. Wang Y, Ji R, Wei X, Gu L, Chen L, Rong Y, Wang R, Zhang Z, Liu B, Xia S: **Esophageal squamous cell carcinoma and ALDH2 and ADH1B polymorphisms in Chinese females**. *Asian Pac J Cancer Prev* 2011, **12**(8):2065-2068.

23. Gu HY, Gong DX, Ding GW, Zhang WB, Liu C, Jiang PC, Chen SC, Chen YJ: **A variant allele of ADH1B and ALDH2, is associated with the risk of esophageal cancer**. *Experimental and Therapeutic Medicine* 2012, **4**(1):135-140.

24. Wu M, Chang SC, Kampman E, Yang J, Wang XS, Gu XP, Han RQ, Liu AM, Wallar G, Zhou JY *et al*: **Single nucleotide polymorphisms of ADH1B, ADH1C and ALDH2 genes and esophageal cancer: A population-based case-control study in China**. *International Journal of Cancer* 2013, **132**(8):1868-1877.

25. Guo YM, Wang Q, Liu YZ, Chen HM, Qi Z, Guo QH: **Genetic polymorphisms in cytochrome P4502E1, alcohol and aldehyde dehydrogenases and the risk of esophageal squamous cell carcinoma in Gansu Chinese males**. *World Journal of Gastroenterology* 2008, **14**(9):1444-1449.

26. Yang SJ, Wang HY, Li XQ, Du HZ, Zheng CJ, Chen HG, Mu XY, Yang CX: **Genetic polymorphisms of ADH2 and ALDH2 association with esophageal cancer risk in southwest China**. *World J Gastroenterol* 2007, **13**(43):5760-5764.

27. Gao Y, He Y, Xu J, Xu L, Du J, Zhu C, Gu H, Ma H, Hu Z, Jin G *et al*: **Genetic variants at 4q21, 4q23 and 12q24 are associated with esophageal squamous cell carcinoma risk in a Chinese population**. *Human Genetics* 2013, **132**(6):649-656.

28. Chen J, Shen B, Lin L, Shi MX, Liu JB: **Polymorphism of DNA repair gene ERCC2/XPD and XRCC1 and its relationship with esophageal squamous cell carcinoma**. *Journal of Modern Oncology* 2015, **23**(23):3429-3432.

29. Chen MR, Wang JM, Guo GP, Hua ZL, Zhou Q, Xu B: **Polymorphism of DNA repair gene XPD and XRCC1 and its relationship with esophageal squamous cell carcinoma**. *Fudan University Journal of Medical Sciences* 2008(02):273-277,281.

30. Wang LZ: **Association of Tagging SNPs in ERCC2Gene with Esophageal Squamous Cell Carcinoma in Henan Han Population**. *Master.* Zhengzhou University; 2012.

31. Xing DY, Qi J, Tan W, Miao XP, Liang G, Yu CY, Lu WF, Zhou CN, Wu M, Lin DX: **Association of genetic polymorphisms in the DNA repair gene XPD with risk of lung and esophageal cancer in a Chinese population in Beijing**. *Chinese Journal of Medical Genetics* 2003, **20**(1):35-38.

32. Zhou RM, Li Y, Wang N, Dong XJ, Zhang XJ, Guo W: **Correlation between single nucleotide polymorphism of DNA repair gene XPD and the risks of esophageal squamous cell carcinoma and gastric cardiac adenocarcinoma**. *Tumor* 2007, **27**(2):118-122,133.

33. Huang CG, Iv GD, Liu T, Liu Q, Feng JG, Lu XM: **Polymorphisms of COMT and XPD and risk of esophageal squamous cell carcinoma in a population of Yili Prefecture, in Xinjiang, China**. *Biomarkers* 2011, **16**(1):37-41.

34. Zhai XD, Mo YN, Xue XQ, Zhao GS, Gao LB, Ai HW, Ye Y: **XRCC1 codon 280 and ERCC2 codon 751 polymorphisms and risk of esophageal squamous cell carcinoma in a Chinese population**. *Bulletin du Cancer* 2009, **96**(10):E61-E65.

35. Zhang L, Wang H, Song ZJ, Zhang XZ: **Impact of single nucleotide polymorphisms in ERCC2 gene and their interaction with smoking on esophageal squamous cell carcinoma risk in Chinese Han population**. *International Journal of Clinical and Experimental Pathology* 2017, **10**(1):730-735.

36. Li RZ, Sun J: **Association between XPD gene polymorphisms and esophageal squamous cell carcinoma**. *Molecular Medicine Reports* 2013, **7**(2):674-678.

37. Liu F: **The association between the AGT/XRCC1 SNPs and the risk of esophageal squamous cell carcinoma in Han population of Northern Sichuan**. *Master.* North Sichuan Medical College; 2014.

38. Song CY, Tan W, Lin DX: **Polymorphisms of DNA repair gene XRCC1 in Chinese population and its relation to esophageal squamous cell carcinoma**. *Chinese Journal of Cancer* 2001, **20**(01):28-31.

39. Yu HJ: **Genotype and Phenotype Study on Cancer Related Genes in Relation to Esophageal Squamous Cell Carcinoma in Yangzhong County, Jiangsu Province, China**. *Master.* Fudan University; 2009.

40. Yun YX, Dai LP, Wang P, Wang KJ, Zhang JY, Xie W: **Association of polymorphisms in X-ray repair cross complementing 1 gene and risk of esophageal squamous cell carcinoma in a Chinese population**. *Biomed Res Int* 2015, **2015**:509215.

41. Liu SP, Zou SJ, Zhao JQ, Zhou BX, Wang ZH, Jia HF, Yuan KS, He JD: **Association between polymorphism of XRCC1 gene and susceptibility to esophageal carcinoma**. *Journal of Practical Oncology* 2013, **28**(03):253-260.

42. Zhao WP, Lu P, Miao ZH, Xu F: **Correlation of XRCC1 Codon 399 Polymorphisms and Risk of Esophageal Squamous Cell Carcinoma in North Henan population**. *Medical Information* 2010, **5**(03):470-472.

43. Feng XX, Li ZF, Wang LB, Zhang JB, Lu ZX: **Study on the relationship between polymorphisms of NQO1 gene and susceptibility to esophageal cancer**. *Chinese Journal of Disease Control & Prevention* 2008, **12**(02):112-114.

44. Li L: **The Correlation between Dietary Factors and NQO1 Gene Polymorphism with Susceptibility to Esophageal Cancer in Southern Fujian Province**. *Master.* Fujian Medical University; 2008.

45. Zhou YL: **Association Research between Environmental Risk Factors, Polymorphism of NQO1 and Susceptibility to Esophageal Cancer**. *Master.* Fujian Medical University; 2005.

46. Yin J, Wang LM, Wang X, Zheng L, Shi YJ, Shao AZ, Tang WF, Ding GW, Liu C, Liu RP *et al*: **NQO1 rs1800566 C > T polymorphism was associated with a decreased risk of esophageal cancer in a Chinese population**. *Scandinavian Journal of Gastroenterology* 2014, **49**(3):317-322.

47. Zhang J, Schulz WA, Li Y, Wang R, Zotz R, Wen D, Siegel D, Ross D, Gabbert HE, Sarbia M: **Association of NAD(P)H: quinone oxidoreductase 1 (NQO1) C609T polymorphism with esophageal squamous cell carcinoma in a German Caucasian and a northern Chinese population**. *Carcinogenesis* 2003, **24**(5):905-909.

48. Duan PF: **A Case-control Study on Polymorphisms of XPA、XPC and Esophageal Carcinoma**. *Master.* Shanxi Medical University; 2007.

49. Huang XX: **Polymorphisms of XPA and XRCC3, Environmental Agent and Susceptibilities to Esophageal, Cardia and Non-cardia Gastric Cancer**. *Master.* Fujian Medical University; 2007.

50. Wan LL, Zhou RM, Wang N, Li Y, Guo W: **Corrolation of XPA polymorphisms to the risk of squamous cell carcinoma and gastric cardia adenocarcinoma**. *Cancer Research on Prevention and Treatment* 2007, **34**(1):63-67.

51. Wang JM: **Genetic and Epigenetic Study on Cancer Related Genes in Relation to Esophageal Squamous Cell Carcinoma**. *PhD.* Fudan University; 2007.

52. Wang XM: **Association Research between Environmental Risk Factors, Polymorphism of MTHFRC677T and Susceptibility to Esophageal Cancer in Xingjian Kazakh**. *Master.* Shihezi University; 2007.

53. Stolzenberg-Solomon RZ, Qiao YL, Abnet CC, Ratnasinghe DL, Dawsey SM, Dong ZW, Taylor PR, Mark SD: **Esophageal and Gastric Cardia Cancer Risk and Folate- and Vitamin B 12-related Polymorphisms in Linxian, China**. *Cancer Epidemiology Biomarkers and Prevention* 2003, **12**(11 II):1222-1226.

54. Zhao P, Lin F, Li Z, Lin B, Lin J, Luo R: **Folate intake, methylenetetrahydrofolate reductase polymorphisms, and risk of esophageal cancer**. *Asian Pac J Cancer Prev* 2011, **12**(8):2019-2023.

55. Song C, Xing D, Tan W, Wei Q, Lin D: **Methylenetetrahydrofolate reductase polymorphisms increase risk of esophageal squamous cell carcinoma in a Chinese population**. *Cancer Res* 2001, **61**(8):3272-3275.

56. Qin JM, Yang L, Chen B, Wang XM, Li F, Liao PH, He L: **Interaction of methylenetetrahydrofolate reductase C677T, cytochrome P4502E1 polymorphism and environment factors in esophageal cancer in Kazakh population**. *World Journal of Gastroenterology* 2008, **14**(45):6986-6992.

57. Qu HH, Cui LH, Wang K, Wang P, Song CH, Wang KJ, Zhang JY, Dai LP: **The methylenetetrahydrofolate reductase C677T polymorphism influences risk of esophageal cancer in Chinese**. *Asian Pac J Cancer Prev* 2013, **14**(5):3163-3168.

58. Chang SC, Chang PY, Butler B, Goldstein BY, Mu L, Cai L, You NC, Baecker A, Yu SZ, Heber D *et al*: **Single nucleotide polymorphisms of one-carbon metabolism and cancers of the esophagus, stomach, and liver in a Chinese population**. *PLoS One* 2014, **9**(10):e109235.

59. Fan XJ, Ren PL, Lu ZJ, Zhao S, Yang XL, Liu J: **The study of esophageal cancer risk associated with polymorphisms of DNA damage repair genes XRCC4 and RAD51**. *Journal of Sichuan University (Medical Sciences)* 2013, **44**(04):568-572.

60. Liu YP, Chen Y, Shi MX, Zhou XM, Mao QH: **Association of single nucleotide polymorphism of DNA damage repair gene RAD51 with esophageal squamous cell carcinoma**. *Journal of Nantong University(Medical Sciences)* 2014, **34**(6):566-568.

61. Zhang SX, Yang S, Xu CQ, Hou RP, Zhang CZ, Xu CP: **Equivocal association of RAD51 polymorphisms with risk of esophageal squamous cell carcinoma in a Chinese population**. *Asian Pacific Journal of Cancer Prevention* 2014, **15**(2):763-767.

62. Xiao Y, Chen W, Cao WK, Chu HJ, Guo W, Pan SD, Jiang J, Dong J, Hu ZB, Shen HB: **Polymorphisms in IL23R gene are associated with risk of esophageal caner in Chinese population**. *Acta Universitatis Medicinalis Nanjing(Natural Science)* 2011, **31**(04):513-516,521.

63. Li M, Yue CL, Jin GQ, Guo HL, Ma HZ, Wang GY, Huang SK, Wu F, Zhao XH: **Rs1884444 variant in IL23R gene is associated with a decreased risk in esophageal cancer in Chinese population**. *Molecular Carcinogenesis* 2019, **58**(10):1822-1831.

64. Chu HJ, Cao WK, Chen W, Pan SD, Xiao Y, Liu Y, Gu HY, Guo W, Xu L, Hu ZB *et al*: **Potentially functional polymorphisms in IL-23 receptor and risk of esophageal cancer in a Chinese population**. *International Journal of Cancer* 2012, **130**(5):1093-1097.

65. Ni B, Chen S, Xie H, Ma H: **Functional polymorphisms in interleukin-23 receptor and susceptibility to esophageal squamous cell carcinoma in chinese population**. *PLoS ONE* 2014, **9**(2).

66. Gao CM, T. T, H. S, Wu JZ, Ding JH, Liu YT, Li SP, Su P, Li ZY, Wang JD *et al*: **The impact of CYP2E1, GSTT1 and GSTM1 polymorphisms on the risk of esophageal cancer**. *China Cancer* 2001, **10**(6):346-349.

67. Wang W, Shi RH, Zhao ZQ: **Impact of CYP2E1 polymorphisms on the risk of esophageal cancer**. *Acta Universitatis Medicinalis Nanjing(Natural Science)* 2004(04):344-347.

68. Liu R, Yin LH, Pu YP: **Association of combined CYP2E1 gene polymorphism with the risk for esophageal squamous cell carcinoma in Huai'an population, China**. *Chinese Medical Journal* 2007, **120**(20):1797-1802.

69. Wang JY, Lin J, Wu J, Cao L, Pei N, Zhang XM: **Case-control study on the correlation between CYP2E1-1239G>C polymorphism and esophagus cancer susceptibility**. *Modern Preventive Medicine* 2016, **43**(20):3822-3826.

70. Shi RH, Wang W, Yu LZ, Huang XY, Chen ZQ, Zhao ZQ: **Polymorphisms in cytochrome P450 2E1 and glutathione transsulfhydrylase P1 genes and susceptibility to esophageal cancer**. *Chinese Journal of Digestive Endoscopy* 2004, **21**(06):392-394.

71. Zhang XJ, Guo W, Wang N, Zhou RM, Dong XJ, Li Y: **The association of MMP-13 polymorphism with susceptibility to esophageal squamous cell carcinoma and gastric cardiac adenocarcinoma**. *Hereditas(Beijing)* 2006, **28**(12):1500-1504.

72. Shi M, Xia JH, Xing HX, Yang WJ, Xiong XY, Pan WT, Han SC, Shang JH, Zhou CC, Zhou LQ *et al*: **The Sp1-mediaded allelic regulation of MMP13 expression by an ESCC susceptibility SNP rs2252070**. *Scientific Reports* 2016, **6**.

73. Chen YZ, Cui XB, Pang XL, Li L, Hu JM, Liu CX, Cao YW, Yang L, Li F: **Relationship between rs2274223 and rs3765524 polymorphisms of PLCE1 and risk of esophageal squamous cell carcinoma in a Kazakh Chinese population**. *Chinese Journal of Pathology* 2013, **42**(12):795-800.

74. Zhang L: **Shaanxi Han Population genetic polymorphisms associated with the risk of esophageal cancer**. *Master.* Northwest University; 2015.

75. Zhou RM, Wang N, Niu CX, Huang X, Huo XR, Li Y: **Association between PLCε1 gene polymorphisms and susceptibility to esophageal carcinoma**. *Chinese Journal of Clinical Oncology* 2014, **41**(22):1437-1441.

76. Duan FJ, Xie W, Cui LH, Wang P, Song CH, Qu HH, Wang KJ, Zhang JY, Dai LP: **Novel functional variants locus in PLCE1 and susceptibility to esophageal squamous cell carcinoma: Based on published genome-wide association studies in a central Chinese population**. *Cancer Epidemiology* 2013, **37**(5):647-652.

77. Gu HY, Ding GW, Zhang WB, Liu C, Chen YJ, Chen SC, Jiang PC: **Replication study of PLCE1 and C20orf54 polymorphism and risk of esophageal cancer in a Chinese population**. *Molecular Biology Reports* 2012, **39**(9):9105-9111.

78. Jia XB, Liu P, Zhang MX, Feng T, Tang HT, Tang ZG, Zhao HQ, Jin TB: **Genetic variants at 6p21, 10q23, 16q21 and 22q12 are associated with esophageal cancer risk in a Chinese Han population**. *International Journal of Clinical and Experimental Medicine* 2015, **8**(10):19381-19387.

79. Hu HC, Yang JM, Sun YH, Yang YJ, Qian J, Jin L, Wang MY, Bi R, Zhang RX, Zhu ML *et al*: **Putatively Functional PLCE1 Variants and Susceptibility to Esophageal Squamous Cell Carcinoma (ESCC): A Case-Control Study in Eastern Chinese Populations**. *Annals of Surgical Oncology* 2012, **19**(7):2403-2410.

80. Guo W, Cui YJ, Fang SM, Li Y, Wang N, Zhang JH: **Association of Polymorphisms of p21cip1 and p27kip1 Genes with Susceptibilities of Esophageal Squamous Cell Carcinoma and Gastric Cardiac Adenocarcinoma**. *Chinese Journal of Cancer* 2006, **25**(02):194-199.

81. Ma YQ: **Association of the p21 and p27 Gene Polymorphisms with Esophageal Squamous Cell Carcinoma**. *Master.* Hebei Medical University; 2006.

82. Liu BT: **Association between SNPs in P53 binding sites and risk of esophageal squamous cell carcinoma**. *Master.* Third Military Medical University of Chinese P.L.A.; 2012.

83. Yang W, Li Y, Ning T, Cai H, Chen Z, Dong Y, Ke Y: **Polymorphisms in the 5' upstream regulatory region of p21(WAF1/CIP1) and susceptibility to oesophageal squamous cell carcinoma**. *Scientific reports* 2016, **6**:22564.

84. Cao YY, Zhang XF, Guo W, Wang R, Ge H, Zhang JH: **Association of the MDM2 polymorphisms with susceptibility of esophageal squamous cell carcinoma and that of gastric cardiac adenocarcinoma**. *Tumor* 2007, **27**(8):628-632.

85. Zhang L, Zhu Z, Wu H, Wang K: **Association between SNP309 and del1518 polymorphism in MDM2 homologue and esophageal squamous cell carcinoma risk in Chinese population of Shandong province**. *Annals of Clinical and Laboratory Science* 2015, **45**(4):433-437.

86. Fu Y: **Association of the Matrix Metalloproteinase-9 Polymorphism with Susceptibility to Esophageal Squamous Cell Carcinoma in Southwest China**. *Master.* Third Military Medical University of Chinese P.L.A.; 2009.

87. Xia P, Chang DM, Dang CX, Meng L, Xue H, Liu Y: **Association between the -1562 C/T polymorphism in the MMP-9 promoter and phenotype of esophageal squamous cell carcinoma in northern Chinese population**. *Academic Journal of Xi'an Jiaotong University* 2010, **22**(1):39-43.

88. Zhang L, Xi RX, Zhang XZ: **Matrix metalloproteinase variants associated with risk and clinical outcome of esophageal cancer**. *Genetics and Molecular Research* 2015, **14**(2):4616-4624.

89. Huang JX, Li X, Wang Y, Lu CM: **Analysis on correlation between multiple single nucleotide polymorphisms loci and risk of esophageal and gastric cancer**. *Chongqing Medicine* 2018, **47**(14):1889-1895.

90. Wang J, Zhang B, Yang Z, Zhou L, Geng T, Li H, Fu X, Xue X, Liu M, Tong R *et al*: **Association of gastrointestinal gland cancer susceptibility loci with esophageal carcinoma among the Chinese Han population: a case-control study**. *Tumour Biol* 2016, **37**(2):1627-1633.

91. Yin J, Wang LM, Zheng L, Wang X, Shi YJ, Shao AZ, Ding GW, Liu C, Chen SC, Tang WF *et al*: **TERT-CLPTM1L Rs401681 C > T Polymorphism Was Associated with a Decreased Risk of Esophageal Cancer in a Chinese Population**. *Plos One* 2014, **9**(7).

92. Wu JZ, Ding JH, Li SP, Gao CM, Zang Y, Zhou JN, Su P, Liu YT, Zhou XF, Wang RH *et al*: **Polymorphisms of Aldehyde Dehydrogenase-2 Genotypes and the Risk of Esophageal Cancer**. *Bulletin of Chinese Cancer* 2001, **10**(12):705-707.

93. Liu P, Zhao HR, Li F, Zhang L, Zhang H, Wang WR, Mao R, Su WP, Zhang Y, Bao YX: **Correlations of ALDH2 rs671 and C12orf30 rs4767364 polymorphisms with increased risk and prognosis of esophageal squamous cell carcinoma in the Kazak and Han populations in Xinjiang province**. *J Clin Lab Anal* 2018, **32**(2).

94. Zhang YG: **SNPs in microRNA Binding Site of EGFR Signaling Pathway and Susceptibility to ESCC**. *Master.* Zhengzhou University; 2014.

95. Xu X, Chen G, Wu L, Liu L: **Association of genetic polymorphisms in PTEN and additional gene-gene interaction with risk of esophageal squamous cell carcinoma in Chinese Han population**. *Dis Esophagus* 2016, **29**(8):944-949.

96. Wang LF, Zhao HG, Li XR, Yi YQ: **FasL gene-844T/C mutation in relation to susceptibility of esophageal cancer in Zhejiang Province**. *Zhejiang Medical Journal* 2013, **35**(20):1791-1794,1801.

97. Zhang M, Wu C, Li B, Du W, Zhang C, Chen Z: **Quantitative assessment of the association between Fas/FasL gene polymorphism and susceptibility to esophageal carcinoma in a north Chinese population**. *Cancer Med* 2016, **5**(4):760-766.

98. Sun T, Miao XP, Zhang XM, Tan W, Xiong P, Lin DX: **Polymorphisms of death pathway genes FAS and FASL in esophageal squamous-cell carcinoma**. *Jnci-Journal of the National Cancer Institute* 2004, **96**(13):1030-1036.

99. Li Y, Zhu WC, Lin ZL: **Correlation between Smoking and the Losing of Gene GSTMI and Esophageal Carcinoma**. *Journal of Practical Medical Techniques* 2004, **11**(03):317-319.

100. Tan W, Song N, Wang GQ, Liu Q, Tang HJ, Kadlubar FF, Lin DX: **Impact of genetic polymorphisms in cytochrome P450 2E1 and glutathione S-transferases M1, T1, and P1 on susceptibility to esophageal cancer among high-risk individuals in China**. *Cancer Epidemiol Biomarkers Prev* 2000, **9**(6):551-556.
